# Determinants and policy approaches to healthcare professional retention in Iran: A mix of scoping review and qualitative evidence

**DOI:** 10.1371/journal.pone.0339855

**Published:** 2026-04-21

**Authors:** Hamideh Keyvani, Reza Majdzadeh, Jalal Hanaee, Leila Doshmangir

**Affiliations:** 1 Department of Health Policy & Management, Tabriz Health Services Management Research Center, School of Management and Medical Informatics, Tabriz University of Medical Sciences, Tabriz, Iran; 2 School of Health and Social Care, University of Essex, Colchester, United Kingdom; 3 Pharmaceutical Analysis Research Center, Tabriz University of Medical Sciences, Tabriz, Iran; 4 Social Determinants of Health Research Center, Department of Health Policy & Management, Tabriz Health Services Management Research Center, School of Management and Medical Informatics, Tabriz University of Medical Sciences, Tabriz, Iran; Flinders University, AUSTRALIA

## Abstract

**Background:**

Ensuring the long-term retention of healthcare professionals has become a central priority for strengthening the resilience and quality of Iran’s health system. A comprehensive understanding of the diverse factors influencing retention, alongside those that may contribute to outward mobility, is essential for informed policymaking. This study adopts a multi-method approach, integrating qualitative inquiry with a scoping review, to explore the determinants shaping healthcare professionals’ decisions to remain in or leave the national health system, the implications for workforce stability, and the effectiveness of current policy responses. By examining both individual and systemic dimensions, and by incorporating insights from migrant professionals, domestic policymakers, and existing evidence, this research contributes a nuanced perspective to the broader discourse on human resources for health in developing contexts. The originality of this study lies in combining first-hand qualitative data with a systematic synthesis of literature, allowing for an in-depth understanding of locally grounded drivers of retention and opportunities for strengthening Iran’s health workforce.

**Methods:**

The research employed a qualitative, multi-phase design. In the first phase, semi-structured interviews were conducted with 15 Iranian healthcare professionals residing in countries such as the United States, Germany, Switzerland, Turkey, and Oman to understand their professional trajectories. In the second phase, interviews with 16 Iranian health administrators, policymakers, and senior professionals were undertaken to capture institutional and policy-level perspectives on retention challenges and opportunities. All interviews were transcribed verbatim and analyzed thematically. Complementing this, a scoping review was conducted to synthesize existing evidence, policy documents, and expert assessments related to the retention and mobility of Iran’s health workforce. Data sources included PubMed, Scopus, and gray literature obtained through institutional repositories and local websites.

**Results:**

Findings highlight several interrelated factors influencing workforce retention. Economic considerations including income stability, purchasing power, and access to research funding remain important determinants of professional commitment. Institutional dynamics such as transparent promotion pathways, supportive management, and opportunities for professional development also play a critical role. Importantly, Iran’s integrated system of medical education and service delivery emerged as a key structural strength, providing early clinical exposure, strong professional identity formation, and continuity between training and practice advantages less commonly available in many other health systems. Participants noted that when financial incentives are combined with accessible professional development programs, equitable workload distribution, and recognition of professional contributions, retention outcomes improve substantially. While destination countries may offer expanded career pathways, the sense of belonging, family ties, and cultural identity were identified as influential factors supporting continued service in Iran. Governmental initiatives including compensation adjustments, training reforms, and targeted retention programs have yielded positive outcomes, though further coordination and sustained implementation are needed.

**Conclusion:**

Strengthening the retention of healthcare professionals in Iran requires a holistic strategy that integrates economic, organizational, educational, and governance reforms. Enhancing remuneration structures, investing in professional development and research capacity, reinforcing the advantages of Iran’s integrated educational service system, and engaging with the healthcare diaspora can collectively support workforce stability. A multi-sectoral, evidence-informed approach has the potential to improve long-term retention, mitigate the need for outward mobility, and promote a more resilient and sustainable health system.

## Introduction

Retaining healthcare professionals is a critical global health challenge with major implications for health systems in both origin and destination countries. Since 2000, the World Health Organization (WHO) has prioritized health workforce stability as a cornerstone of global health policy. The Global Strategy on Human Resources for Health: Workforce 2030 outlines key objectives, such as strengthening health workforce data systems, enhancing governance, and promoting equitable geographic distribution of personnel [1,2]. This framework emphasizes coordinated, evidence-based strategies to mitigate shortages and inequities, which are essential for achieving Universal Health Coverage (UHC) and the Sustainable Development Goals (SDGs).

At the regional level, the Eastern Mediterranean Regional Strategy for Health Workforce Development (2017–2030) highlights priorities including workforce expansion, gender equity, and improved retention. It encourages member states to implement evidence-based policies that bolster workforce capacity and counteract emigration pressures [3]. These priorities gained further prominence at the recent Eastern Mediterranean Regional Meeting on Health Workforce in Qatar, where stakeholders advocated for collaboration, knowledge sharing, and innovative retention strategies [4]. Together, these efforts highlight the importance of aligning national policies with global and regional standards to ensure sustainable and equitable healthcare delivery.

Iran’s experience within this global and regional context offers valuable insights into health workforce retention in middle-income countries. Iran has established a robust tradition in medical education and public health, achieving notable advancements such as expanded postgraduate medical training programs and improved primary healthcare access [5,6]. These accomplishments have strengthened the health system, enabling effective responses to challenges like the COVID-19 pandemic through the Healthcare Incident Command System [7]. Despite these successes, Iran faces ongoing emigration of healthcare professionals, which can strain equitable access, workforce stability, and overall system performance [8].

Iran’s health workforce policies largely align with WHO and regional guidelines, yet gaps persist in addressing the interplay of individual, professional, and systemic factors influencing retention. These include the effectiveness of current strategies and the long-term effects of mobility on healthcare quality, education, and governance [9]. Opportunities exist within Iran’s Health Transformation Plan (HTP), which has enhanced service coverage and financial protections, creating a foundation for further retention efforts [10,11]. Addressing these areas is vital for developing context-specific, sustainable approaches that enhance retention and support global health resource goals.

Official data indicate that approximately 1.9 million Iranian migrants, representing about 2.2% of the national population, were living abroad in 2020. Among these migrants, around 8% were healthcare professionals, including physicians, nurses, and allied health worker [12,13]. This estimate may understate the total, excluding dual citizens and long-term residents. Primary destinations include the United States, Canada, Germany, Sweden, and France [14]. Emigration has risen steadily over the past three decades, reflecting demographic and socioeconomic shifts. Following post-revolutionary reforms in education and healthcare, medical professional outflows have increased. In 2022, the International Organization for Migration (IOM) identified Iran among the countries with substantial outflows of highly educated professionals in health and academia [15]. The WHO projects a global shortage of 18 million health workers by 2030, with Iran contributing to this through skilled emigration [16]. Estimates suggest 10,000 Iranian medical specialists leave annually, mainly for North America and Europe [17]. Surveys show 52.5% of healthcare professionals intend to emigrate, aligning with findings that 54.7% exhibit such tendencies [18,19]. These patterns challenge Iran’s health system by widening service disparities, creating shortages, and increasing access inequities, as seen in other low- and middle-income countries [20]. Beyond these aggregate migration trends, Iran’s health system has evolved under prolonged international sanctions that have affected macroeconomic conditions and placed additional pressure on health-sector capacity. Empirical studies and UN-mandated assessments show that sanctions and financial over-compliance disrupt imports of medicines and medical technologies, raise prices, and reduce the availability of essential drugs and supplies, particularly for chronic and life-threatening conditions. These constraints may lead some households to delay or forgo recommended care and have been associated with adverse health outcomes, with gender and socioeconomic differences in vulnerability [21,22]. For healthcare professionals, delivering care in this context of resource constraints and limited policy flexibility can increase moral distress and narrow opportunities for professional development and international collaboration, so that outward migration may be perceived as one possible response to accumulated professional and personal pressures. The recent UN Security Council snapback under Resolution 2231, which has reimposed multilateral sanctions on Iran, has renewed concerns that these pressures on access to medicines and essential services may intensify further unless robust health safeguards are implemented [23].

Research indicates that economic and professional considerations are among the key factors influencing emigration intentions among Iranian medical students and health professionals. In particular, opportunities for career development and financial stability are frequently highlighted, with one study reporting that 70% of respondents identified economic factors as influential in their decision-making [24,25]. Perceptions of workplace inequity and job dissatisfaction also contribute to these intentions [9,26]. Beyond structural and professional conditions, socio-political and psychological dimensions; such as concerns about personal and family well-being, occupational stress, and burnout; play a notable role [25,27,28]. The challenges faced by the health workforce were further amplified during the COVID-19 pandemic, which increased workloads and emotional strain, and in some cases accelerated decisions to seek opportunities abroad [29]. Additionally, turnover within academic medicine remains a concern, as nearly one-quarter of faculty members reportedly leave their positions, potentially affecting the continuity of education and mentorship [30].

Strategic workforce planning is essential to counter these trends and sustain system continuity [31]. Sustainable retention requires addressing core issues like equity, adequate compensation, and supportive institutional structures [26,28]. Iran’s system fosters retention through initiatives such as the HTP’s remuneration improvements and research exchange programs, which enhance professional satisfaction and institutional appeal [10,32,33]. Factors promoting retention include equitable organizational justice, job satisfaction, and opportunities for career growth, which reduce turnover intentions [26,28,34]. Programs like the Human Resources for Health initiative by the WHO Regional Office further support capacity building and retention in Iran [35]. Although some interventions, such as health tourism and elite attraction strategies, show promise, they require broader implementation [32,33].

Previous studies on Iran’s health workforce have focused mainly on push and pull factors, with less attention to policy effectiveness or comprehensive solutions [36–38]. This study addresses these gaps by combining empirical data and policy analysis to examine retention determinants, system impacts, and current policy outcomes.

To offer a multi-level explanation of healthcare workforce retention, this study employs an integrated theoretical framework spanning macro, meso, and micro levels.

At the macro level, World Systems Theory [39] elucidates global economic influences. It posits that inequalities between high-income (“core”) and low- to middle-income (“peripheral”) countries drive skilled labor flows toward developed systems, explaining the draw of Iranian professionals to advanced economies. At the meso level, the Health Workforce Dynamics Framework [2] examines institutional and systemic aspects. It addresses push factors (e.g., resource limitations, workload pressures, policy constraints) and pull factors (e.g., professional opportunities, fair pay, secure environments) within Iran’s health system. At the micro level, Behavioral Economics and the Theory of Planned Behavior [40] inform individual decisions. These theories account for attitudes (e.g., perceived migration benefits), subjective norms (e.g., family or peer influences), and perceived control (e.g., information or visa access) shaping retention choices. This integration provides a theoretically robust analysis of how global structures, institutional contexts, and personal motivations interact to influence workforce stability in Iran.

Building on this framework, we apply a mixed-methods design that brings together qualitative interviews with emigrated and domestic stakeholders and a scoping review of empirical and policy literature. The study aims to identify multilevel determinants of healthcare professional retention in Iran and to examine how existing policies shape mobility and workforce stability. By linking lived experience with documentary evidence, we generate practical recommendations for strengthening retention strategies in Iran and, by extension, in comparable middle-income settings.

## Methods

This study adopted a mixed-methods approach, integrating qualitative interviews with a scoping review of the existing literature. To ensure methodological rigor, transparency, and reproducibility, the research was conducted in accordance with established reporting standards, specifically the Consolidated Criteria for Reporting Qualitative Research (COREQ) [41] and the Preferred Reporting Items for Systematic Reviews and Meta-Analyses extension for Scoping Reviews (PRISMA-ScR) [42].

- Qualitative Phase

The qualitative phase involved semi-structured interviews with 31 participants. This group included 15 Iranian healthcare professionals who had emigrated to destinations such as the United States, Germany, Switzerland, Turkey, and Oman (comprising 4 physicians, 4 nurses, and 7 health researchers). An additional 16 participants were healthcare experts and policymakers based in Iran (including 3 physicians, 3 administrators, and 10 university faculty members). Recruitment occurred through professional and academic networks, with purposive sampling to select individuals who had at least one year of migration experience and expressed willingness to discuss their perspectives.

Data collection took place from April 2024 to May 2025, using flexible communication methods such as video calls, telephone interviews, voice messages, or text-based exchanges, depending on participants’ preferences and locations. Researchers obtained verbal informed consent at the start of each session, which they recorded. The consent explanation covered confidentiality, voluntary participation, and the right to withdraw without repercussions.

Interviews lasted 30–45 minutes and continued until data saturation occurred, marked by the absence of new themes. A semi-structured guide (S1 Appendix) directed the discussions, featuring open- and closed-ended questions on migration motivations, professional and personal influences, challenges in host countries, policy perceptions, and retention or return recommendations. A trained qualitative researcher (the first author) conducted all interviews to maintain consistency and neutrality, minimizing bias. Reflexive journaling after each session documented the researcher’s reflections, while member checking; sharing summaries for participant feedback; verified interpretive accuracy.

Analysis followed Braun and Clarke’s six-phase thematic approach [43]. Researchers generated inductive codes from transcripts, then grouped them into themes such as push-pull factors, policy responses, and outcome perceptions. Manual coding occurred in Microsoft Excel for detailed text engagement. Reporting aligned with the COREQ checklist (Consolidated Criteria for Reporting Qualitative Research) [41] to uphold standards of qualitative inquiry.

- Scoping Review Phase

The scoping review followed the PRISMA-ScR guidelines (Preferred Reporting Items for Systematic Reviews and Meta-Analyses extension for Scoping Reviews) [42] to map and synthesize evidence on healthcare workforce retention in Iran, including policies and institutional documents. Search terms encompassed “retention,” “health workforce,” “brain drain,” “migration”, “emigration”, “human resources for health,” and “health policy,” along with Persian equivalents. Databases included PubMed, Scopus, Web of Science, Google Scholar, Scientific Information Database (SID), and Magiran. Additional sources comprised repositories from the WHO, Iranian Ministry of Health and Medical Education, International Organization for Migration (IOM), and Iran Migration Observatory. Searches spanned February to May 2025, targeting publications from the past 50 years.

Inclusion criteria focused on English- or Persian-language materials addressing retention of Iran’s healthcare workforce or related policies, with empirical or contextual relevance. Researchers excluded non-credible sources, irrelevant documents, and inaccessible full texts. Two independent reviewers screened records in two phases: title and abstract review, followed by full-text evaluation. They extracted key data, such as policy type, objectives, target groups, mechanisms, and outcomes, into a matrix for thematic synthesis. This process identified patterns in policies and implementation, interpreted through the study’s conceptual framework to highlight gaps, strengths, and future directions.

**Fig 1:**
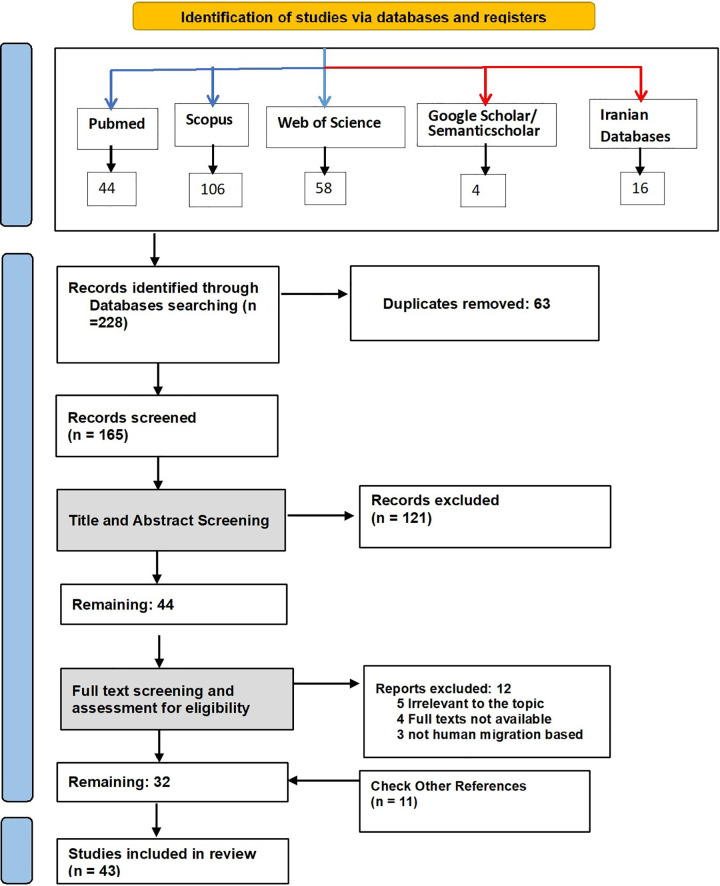
PRISMA flow diagram illustrating the identification, screening, eligibility, and inclusion process for peer-reviewed empirical studies.

**Fig 2:**
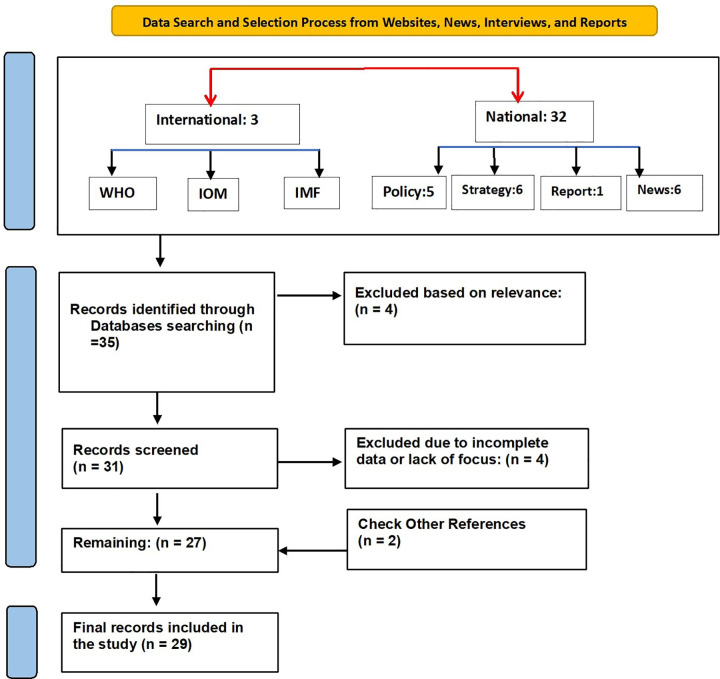
PRISMA flow diagram showing the identification and selection of institutional and policy documents (e.g., government reports, WHO publications, and policy briefs) related to workforce retention strategies.

Two PRISMA flow diagrams visualized the process: Fig 1 for peer-reviewed articles (detailing identification, screening, and exclusion reasons); Fig 2 for institutional documents (e.g., government reports, WHO publications, policy briefs) that informed national strategies and interventions. This dual structure provided a thorough examination of academic and policy evidence on workforce retention.

### Search process

The initial search identified 263 records: 228 from electronic databases and 35 from institutional sources or local websites. After deduplication, 200 unique items underwent title and abstract screening for relevance to retention factors, strategies, and outcomes. Full-text review then excluded materials with insufficient healthcare focus, methodological limitations, or lack of emphasis on workforce retention. In total, 72 sources met the inclusion criteria and contributed to the synthesis: 43 peer-reviewed articles and 29 institutional or policy documents.

### Data extraction and categorization

Researchers used a standardized extraction form to compile data, organizing it into three domains: retention and migration drivers (economic, professional, and socio-political factors), intervention strategies (policy initiatives and institutional measures), and effectiveness evaluations (impacts on retention or return). Two independent reviewers conducted extraction and thematic categorization. They resolved discrepancies through team discussions, promoting reliability and validity. This process ensured accurate interpretation of scholarly and policy evidence. S1 and S2 Tables summarize the extracted data and categorized findings.

### Ethics statement

The study received ethical approval from the Research Ethics Committee of Tabriz University of Medical Sciences (IR.TBZMED.REC.1402.856). It followed principles for human participant research, addressing three core elements:

- Informed Consent: Researchers explained study objectives, procedures, risks, benefits, and participant rights. They obtained verbal informed consent before each interview, recorded for documentation.
- Confidentiality: Anonymity and privacy remained paramount. Identifying details were omitted, and pseudonyms protected quoted material.
- Voluntary Participation: Individuals joined freely, with clear information on withdrawal rights at any stage without consequences.

These measures upheld participant autonomy, research integrity, and transparency.

## Results

This study analyzes the determinants of healthcare professional retention in Iran, evaluates existing interventions, and proposes evidence-based strategies. It explores interactions among economic, social, and governance factors that influence workforce stability. Data derived from 72 sources; 43 peer-reviewed articles and 29 institutional or policy documents, complemented qualitative interviews with 15 emigrated Iranian healthcare professionals and 16 domestic experts and policymakers. The mixed-methods design yielded a multifaceted assessment of retention drivers and their effects on Iran’s health system.

S1 Table synthesizes literature-extracted data, including study characteristics, emigration and retention factors, and policy recommendations[9,16,18,19,24–29,31,34,36,38,44–71]. S2 Table summarizes institutional and policy documents, detailing challenges, impacts, and responses, with specifications on issuing organizations, document types, authorship, target groups, and policy objectives[10,30,72–96]. S3 Table presents the thematic framework derived from the qualitative analysis, comprising eight overarching categories and associated subthemes. These are substantiated with participant quotations that illuminate diverse perspectives on retention dynamics. S4 Table shows the annual distribution of publications on Iran’s healthcare workforce retention and emigration over the past decade, reflecting growing scholarly interest. S5 Table aggregates key migration factors from the literature, listing the number of citing articles and references for each category (e.g., economic, political, professional); multiple factors in single studies result in overlapping citations.

The following sections detail findings on retention determinants, impacts of emigration, policy evaluations, and innovative strategies for workforce stability and reintegration.

### Publication trends and thematic overview

S4 Table reveals fluctuations in publication volume on healthcare workforce retention and migration in Iran, with a recent upsurge (e.g., 11 articles in 2023; six each in 2018, 2022, and 2024). S5 Table highlights economic factors as most prevalent (23 studies), followed by organizational and administrative elements (16 studies), social and cultural influences (14 studies), and political aspects (11 studies) [97,98]. Educational, structural, occupational, and psychological factors appeared less frequently. These counts reflect the 43 included peer-reviewed articles.

From 2018 to 2019, research emphasized general migration patterns in Iran, with minimal attention to healthcare-specific retention. The COVID-19 pandemic (2020–2021) spurred studies on health systems and workforce mobility, amplifying focus on retention amid global disruptions and shortages. Publications peaked in 2022–2023, driven by economic decline and intensified sanctions. Between 2023 and 2024, policy briefs and evaluations increasingly connected workforce shortages to emigration, fostering evidence-informed discussions.

Literature consistently identified economic instability, professional dissatisfaction, and social pressures as core retention challenges. Integrating interview and review data, five thematic domains emerged: economic and financial factors for stability; professional development and educational factors; organizational and workplace factors; socio-cultural and psychological factors; and political and geopolitical factors. These domains reveal how individual, organizational, and systemic elements interact to shape retention patterns, influencing Iran’s health system and socio-economic framework.

### Root causes of retention challenges

#### Economic and financial factors.

Economic considerations constitute an important dimension influencing the retention of healthcare professionals in Iran. Evidence from studies and qualitative accounts indicates that income levels, inflation, and the perceived balance between workload and compensation shape decisions regarding continued service within the national health system [18,19,26,29,40]. Policy document analysis corroborates these insights, revealing that health workforce policies often overlook real wage erosion from inflation and economic volatility [14,24,28,35]. Many participants emphasized that maintaining a reasonable standard of living and receiving financial recognition aligned with their expertise contributes positively to their commitment to remain in the workforce [24,28,99].

While comparisons with compensation packages in some countries were raised as reference points, several respondents also highlighted the value of economic stability and social security embedded within Iran’s public health system. One participant stated: “After years of experience, my salary in Iran was a fraction of what professionals earned abroad for similar work, which made migration an attractive option for career advancement “(P6, male nurse, migrated to Oman). For many healthcare professionals, the predictability of employment in the public sector, access to subsidized benefits, and long-term career pathways serve as important incentives for retention [72].

Funding availability for research and innovation was identified as another factor influencing professional satisfaction. Some participants felt constrained by limited funding, while others emphasized that national programs have increasingly supported research capacity-building:

“Although resources can be limited, recent funding mechanisms opened valuable opportunities for advancing my projects.” (P12, female researcher, Iran)

Over recent years, the Iranian government has implemented several policies aimed at strengthening financial incentives and supporting workforce retention. These include adjustments to clinical service tariffs, targeted payments for high-demand specialties, and revisions of service valuation [3,100]. Such changes have contributed to improving income stability and promoting continued service, particularly among specialists and experienced nurses.

Salary adjustments, bonuses, and monetary allowances remain core components of national retention strategies [10,73]. Importantly, participants noted that financial incentives are most effective when paired with broader opportunities for professional development and supportive organizational conditions. Bureaucratic hurdles and institutional under-support similarly fueled frustration. Another noted: “The exhausting and demoralizing administrative processes for research funding and career advancement pushed many of us to look for simpler systems abroad” (P9, male researcher, migrated to Turkey). Additionally, diversification efforts, such as expanding health tourism, have been proposed as promising, long-term strategies to generate revenue that can further enhance retention initiatives and support financial sustainability [32].

National reforms, including the HTP, have not only improved access to care across regions [31] but have also contributed to more structured compensation packages and reinforced financial incentives. Overall, the findings suggest that economic strengthening, combined with supportive policy measures, fosters greater job satisfaction, enhances professional stability, and contributes positively to the long-term retention of healthcare professionals in Iran.

#### Professional development and educational factors.

Professional development and educational structures significantly shape retention within Iran’s health workforce. Although rapid expansion in training programs occasionally outpaced investments in educational infrastructure, Iran’s unique model of integrated medical education and healthcare service delivery remains a notable strength [101]. This integration; which is not commonly seen in many countries; creates opportunities for learners to gain early clinical exposure, aligns education with service needs, and supports smoother transitions into the workforce. Participants acknowledged the value of this model in developing applied skills and fostering early professional identity.

Some participants commented that curricula in certain fields still lean toward theoretical instruction. Yet others highlighted that the integrated educational–service system has substantially improved practical training opportunities across medical universities:

“Because education and service delivery are integrated, our students gain real clinical experience much earlier than in many other countries.” (R14, manager, Iran)

Access to research opportunities and advancement pathways was also identified as influential. While some participants pointed to challenges, others emphasized that national initiatives have expanded opportunities significantly. Programs such as the Physician-Researcher Track and the National Institute for Medical and Health Research Development (NIMAD) have enabled researchers and clinicians to obtain competitive grants, participate in collaborative projects, and gain specialized training [34,45].

Participants acknowledged both the achievements and the areas requiring further expansion:

“Some research development programs created real growth opportunities for many of us, and expanding these programs could encourage more colleagues to remain in the system.” (R2, manager, Iran)

Strengthening research infrastructure, fostering innovation funding, enhancing laboratory capacity, and promoting international academic collaborations were among the proposed strategies for supporting career growth. Many participants also noted that improving academic autonomy and clarifying promotion pathways would further encourage retention. Importantly, respondents believed that investments in educational quality and research capacity can even attract Iranian professionals abroad to return:

“Providing competitive research environments can both retain current professionals and encourage those abroad to return.” (P15, male researcher, Switzerland)

Collectively, these findings highlight that Iran’s distinctive educational strengths, coupled with targeted academic development programs, can play a pivotal role in improving healthcare professional retention.

#### Organizational and workplace factors.

Organizational dynamics and workplace conditions play a central role in shaping healthcare professionals’ decisions to remain within the Iranian health system. Across included studies, participants emphasized the importance of institutional support, balanced workloads, transparent promotion pathways, and fair task distribution [26,28,44,72]. Transparent governance and merit-based advancement were repeatedly identified as factors that strengthen a sense of fairness and long-term commitment [100].

Workplace conditions in rural and underserved regions were noted as particularly influential [101]. Many participants referred to challenges related to workload and resource distribution. However, others also recognized that rural practice provides meaningful opportunities for professional growth, community engagement, and expanded clinical autonomy:

“Serving in a smaller community allowed me to use a broader range of skills and develop deeper relationships with patients.” (P5, female nurse, Iran)

Targeted organizational strategies, such as improving workload distribution, establishing supportive supervision systems, enabling flexible scheduling, and strengthening procedural transparency, have shown positive impacts on morale and retention [102]. Participants emphasized that recognition, acknowledgment of effort, and participatory decision-making significantly reinforce commitment:

“When workload is fairly distributed and one’s efforts are acknowledged, it strengthens our motivation to stay.” (P8, female nurse, USA)

Mandatory rural service programs were described as essential for ensuring equitable national service coverage [103]. Although some participants viewed these policies as restrictive, others highlighted that structured rural service allows early-career professionals to gain valuable experience and fosters strong professional identity, particularly when accompanied by adequate support systems.

Overall, findings indicate that organizational reforms emphasizing fairness, recognition, and supportive workplace cultures can meaningfully enhance professional satisfaction, strengthen national service commitment, and contribute to the retention of the healthcare workforce in Iran.

#### Socio-cultural and psychological factors.

Sociocultural and psychological dimensions strongly influence healthcare professionals’ commitment to remain in the national health system [25,29,46]. Participants highlighted the importance of professional recognition, ethical practice environments, and emotional well-being. During the COVID-19 pandemic, recognition of healthcare workers’ contributions became particularly visible, reinforcing a sense of collective responsibility and national appreciation [25].

Many participants emphasized that acknowledgment and respect enhance job satisfaction and strengthen their desire to continue serving:

“It’s not just about money, being valued and respected motivates us to stay.” (P2, male doctor, USA)

Another doctor added: “Almost everyone I know has left or is planning to leave. Staying feels like giving up on growth. The outside world simply offers recognition and opportunities that feel unattainable here” (P1, doctor, Germany).

Ethical alignment and professional integrity were also influential. Participants noted that environments supporting ethical practice and professional autonomy contribute positively to retention.

Family ties, cultural identity, and a strong sense of belonging emerged as key motivations for remaining in or returning to Iran:

“Being close to family and connected to my cultural roots is a major factor in staying.” (P4, male physician, Iran)

Supportive environments for women in healthcare were another important dimension. Participants noted that policies addressing gender equity, work–life balance, and inclusive leadership pathways strengthen women’s engagement and long-term commitment [59,104]:

“When female professionals feel heard and supported, their commitment to stay grows significantly.” (R16, female official, Iran)

Overall, these findings suggest that enhancing professional dignity, ensuring ethical practice environments, strengthening family and cultural connections, and supporting gender-inclusive policies can reinforce retention and contribute to a committed national health workforce.

#### Political and geopolitical factors.

Political and geopolitical conditions, including the impact of international sanctions, form an important contextual factor shaping healthcare professionals’ experiences in Iran. Participants noted that sanctions influence access to certain technologies, medical supplies, and international collaborations. However, many also highlighted that, despite these constraints, Iran has maintained a resilient health system with strong domestic training capacity and internal expertise. The cumulative effects of sanctions; ranging from supply shortages to funding barriers; have intensified economic strain and operational difficulties within the healthcare sector. These findings are consistent with prior research demonstrating that sanctions undermine healthcare infrastructure and contribute to workforce attrition [22,105,106].

Some participants acknowledged challenges in resource availability, while others emphasized that local innovation and domestic production capacity have grown substantially in response, creating opportunities for scientific advancement and professional engagement:

“Despite limitations, local research groups have developed strong capacities, and domestic production is improving each year.” (P3, male physician, Iran)

International frameworks such as the WHO Global Code of Practice [74,107] offer guidance for managing health workforce mobility. While geopolitical constraints shape how such frameworks are applied, national strategies continue to emphasize ethical recruitment and the strengthening of internal retention systems.

Strengthening engagement with the healthcare diaspora was identified as an effective approach for promoting “brain circulation.” Establishing a national diaspora engagement office could facilitate collaborative research, mentorship programs, and short-term academic return visits, allowing expatriate professionals to contribute to national capacity-building [73,47]:

“A structured platform for diaspora collaboration could greatly support domestic scientific development.” (R13, supervisor, Iran)

Flexible models, including shared research appointments, virtual academic networks, and short-term visiting positions, were noted as practical mechanisms for maintaining scientific exchange under geopolitical constraints:

“Flexible collaboration mechanisms, such as virtual research networks, would encourage my continued involvement.” (P14, researcher, England)

Comparative evidence from countries such as India, China, and Canada demonstrates that merit-based systems, structured reintegration pathways, and supportive scientific exchange platforms can enhance retention while promoting global collaboration [108–110]. These insights underscore the potential of integrating diaspora collaboration strategies with national workforce planning to reinforce professional engagement and encourage sustained participation within Iran’s healthcare system [108].

### Consequences of healthcare workforce emigration

Emigration of healthcare professionals yields two principal effects: shortages of clinical staff, especially in rural and underserved areas, and erosion of institutional and intellectual resources. Many facilities operate with reduced personnel, threatening service continuity and patient care quality. An Iranian physician and researcher stated: “Clinics are understaffed, risking vulnerable populations” (R3, physician/researcher, Iran). A female administrator added: “The departure of doctors and nurses has left many rural clinics short-staffed, making it difficult to provide vital healthcare services” (R6, administrator, Iran).

These shortages coincide with productivity declines, as experienced staff losses impair service delivery. An administrator noted: “The migration of experienced professionals has created some challenges in maintaining specialized capacity across treatment, diagnostic, and management services “ (R7, female professor, Iran). Analysis of ten institutional and research documents confirms a direct link between emigration and diminished healthcare access, alongside productivity reductions in essential areas [44,48,49,72,75–80].

While long-term migration may reduce the availability of skilled professionals in Iran, it can also influence the pace of innovation and research development. Several studies have discussed the potential implications of talent mobility for national scientific capacity and research competitiveness [18,49,50–55]. A specialist observed: “ The movement of researchers and scholars abroad has, at times, made it challenging for domestic institutions to sustain the same level of research momentum “ (R12, specialist, Iran).

A nurse working in the UAE reflected: “Some of our highly experienced professionals have migrated. Although the new generation is committed and capable, maintaining mentorship and clinical leadership requires continuous investment in professional development” (P7, male nurse, UAE).

These trends highlight the importance of policies that strengthen knowledge exchange, collaboration, and capacity-building between domestic and international professionals.

### Assessment of current retention policy effectiveness

Policy evaluation across four dimensions; effectiveness, cost, coverage, and sustainability; reveals limited success of existing retention initiatives (see S6 Table).

#### Economic incentives.

Financial strategies, such as salary enhancements and bonuses, form the basis of five national policies [10,73,81–83]. Four empirical studies [18,49,56,57] and participant insights indicate modest impacts, undermined by inflation and fiscal limitations. One participant observed: “Bonuses do not address root causes” (P1, male physician, Germany). These measures favor urban professionals and falter amid economic instability. Another respondent noted: “The increases in basic salaries and random bonuses do not improve our long-term financial situation or career prospects enough to justify staying” (R13, manager, Iran).

#### Talent retention programs.

Initiatives like the Physician-Researcher Program and the National Institutes for Medical and Health Research Development offer moderate career advancement benefits [9,33,34,45]. Scalability and funding continuity pose challenges. A manager reflected: “Programs like NIMAD gave me hope to stay, but I saw so many qualified colleagues left out because the funding couldn’t stretch further. Sustainable support is essential if we are serious about keeping talent here” (R2, manager, Iran).

#### Economic diversification and health tourism.

Two studies position health tourism as a means to bolster sector finances [32,80]. Leveraging Iran’s strengths in procedures like organ transplantation and infertility treatment, these efforts create revenue to offset attrition. Beladi et al. (2015) [32] stress that global competitiveness requires investments in quality and infrastructure, constraining immediate viability.

#### Health transformation plan.

This plan has narrowed regional disparities, as evidenced in four studies [10,11,31,34]. Administrative complexities and fiscal burdens hinder execution, casting doubt on long-term viability without robust planning.

#### Workplace reforms and mandatory service.

Efforts to promote workplace equity yield incremental retention gains [9,10,26,83], tempered by bureaucratic delays. Mandatory rural service, however, backfires. A physician stated: “Forced rural service fueled my resolve to leave” (P2, male doctor, USA). Such mandates exhibit low efficacy and elevate social costs, intensifying early-career discontent.

#### Alignment with global frameworks.

The WHO’s Global Code of Practice on the International Recruitment of Health Personnel [74,107] provides ethical guidelines for the migration of health workers. In Iran, factors such as international sanctions and certain administrative and financial limitations have posed challenges to the full implementation of this code. These circumstances, often beyond the country’s direct control, can affect international cooperation and access to some resources, making the application of the Code more complex.

Overall, Iran’s retention policies suffer from disjointed execution, funding shortages, and short-term focus, reducing their ability to counter complex emigration drivers.

### Proposed strategies to enhance retention and facilitate return

#### Economic and professional reforms.

Studies recommend aligning healthcare professionals’ compensation with international and regional benchmarks, alongside investments in research infrastructure [9,18,24,31,44,49,52,56,58,59]. Competitive packages and advanced facilities could retain talent and encourage repatriation. A participant noted: “Offering competitive salaries alongside advanced research environments could serve both to retain professionals and to attract those working overseas back to Iran” (P15, male researcher, Switzerland).

Expanded funding for research, innovation, and technology transfer would promote professional satisfaction and local advancement opportunities. Policy documents also endorse medical tourism to generate revenue for sector sustainability [2,104]. Infrastructural limitations and ethical issues, such as prioritizing international patients amid domestic demands, temper this approach [86]. An interviewee observed: “While health tourism holds promise given our expertise, prioritizing foreign patients over local populations is problematic when facilities are already overstretched” (R5, manager, Iran).

#### Governance reforms.

Eight studies emphasize governance improvements through transparency, accountability, and merit-based systems to restore trust and reduce emigration [9,18,26,28,106,83,58,59]. Streamlined bureaucracy and institutional autonomy would enhance efficiency and morale. A participant stated: “Implementing transparent, merit-based promotion systems and reducing bureaucratic hurdles would incentivize professionals to remain committed to national service” (R14, manager, Iran).

Regional decentralization supports tailored policymaking for diverse contexts. An official remarked: “A uniform policy approach is ineffective in a country as diverse as Iran. The challenges faced by underserved provinces differ markedly from those in major urban centers. Empowering local institutions fosters a sense of recognition and value among professionals” (R15, male official, Iran).

Participatory leadership, involving professionals in decisions, would strengthen engagement and loyalty. A supervisor highlighted: “Professionals depart not solely due to financial reasons but because they feel like interchangeable parts. Retention requires inviting them to actively participate in shaping the system’s future rather than merely executing directives” (R11, male supervisor, Iran).

#### Diaspora engagement.

Five policy documents advocate creating a dedicated Diaspora Engagement Office to coordinate collaborations with Iranian healthcare professionals abroad [73,47,48,84,85]. This entity would support joint research, mentorship, short-term fellowships, and knowledge exchanges. A supervisor stated: “A diaspora office would enable expatriates to contribute their expertise to domestic projects, thereby fostering innovation and development” (R13, supervisor, Iran).

This model mirrors successful precedents, such as India’s structured diaspora initiatives, which have advanced medical and scientific progress through knowledge transfer [111]. An official emphasized: “Concerns should not be limited to the physical emigration of our most talented individuals; rather, the focus must be on maintaining robust connections to ensure that their innovations and expertise continue to benefit Iran” (R9, male official, Iran).

#### Workplace reforms.

Uneven workload distribution and inadequate conditions, especially in rural areas, contribute to burnout and attrition. Equitable task allocation, improved staffing ratios, and supportive oversight promote retention. A 2022 study showed that workload redistribution reduced turnover intentions by 20% among nurses and physicians [112]. A 2024 analysis linked burnout to compromised care quality and patient satisfaction [113]. A nurse observed: “When workload is fairly distributed and one’s efforts are acknowledged; it significantly impacts morale. It fosters a sense of being valued rather than exploited” (P8, female nurse, USA).

Human resource policies, including flexible schedules, mental health support, and participatory planning, could further elevate morale and retention (see S3 Table).

#### Family and cultural connections.

Four studies identify cultural identity reinforcement and family reunification as return migration incentives [47,48,49,114]. A physician noted: “Proximity to family and reconnecting with my cultural roots would inspire me to return and rediscover my identity in Iran” (P4, male physician, Belgium).

Current return narratives often center on loss: “Regrettably, for many, the primary impetus for returning home is to attend funerals of loved ones. Return pathways should instead be constructed around hope, collaboration, and meaningful engagement rather than solely grief” (P12, male academic, Spain). Initiatives promoting cultural affiliation, professional reintegration, and family incentives would encourage voluntary returns and ongoing involvement.

#### Social and cultural strategies.

Twelve studies stress restoring professional dignity and public esteem to curb emigration [1,2,16,21,28,30,73,81,83,56,112,113]. Public recognition, policymaking inclusion, and ethical workplace reforms would cultivate pride [95]. A nurse remarked: “Recognition of our efforts cultivates a sense of belonging, thereby diminishing the inclination to seek opportunities abroad” (P8, female nurse, USA).

Addressing gender inequities and social barriers, particularly for women, aligns with strategies in middle-income countries [109,110]. An official shared: “What truly encouraged me to remain was not financial incentives, but the sense of respect and equal opportunity within my team. When women feel heard, supported, and valued, it changes their outlook on staying versus leaving” (R16, female official, Iran).

#### Global collaboration and flexible return pathways.

International partnerships, via conferences, fellowships, and joint projects, allow expatriates to contribute without full repatriation. A researcher stated: “Engagement in global projects allows me to maintain connections with Iran while advancing my discipline” (P13, researcher, USA).

This resembles China’s Thousand Talents Plan, which has engaged over 7,000 experts through flexible mechanisms [115]. Another researcher noted: “I am eager to contribute to Iran’s healthcare system; however, permanent return is currently unfeasible. Flexible programs, such as joint research appointments, would encourage my participation without hesitation” (P14, researcher, England). Hybrid models blending mobility and collaboration could offset brain drain and strengthen global scientific ties.

### Insights from international policy frameworks

The WHO’s Global Code of Practice on the International Recruitment of Health Personnel and the Global Compact for Migration offer ethical guidelines to support sustainable health workforce management and mitigate migration’s negative effects on origin countries [74,116–118]. These instruments promote international cooperation, fair recruitment, and evidence-based retention strategies to protect source-country health systems.

Although non-binding, their implementation has advanced monitoring, bilateral agreements, and retention programs in low- and middle-income countries (LMICs). For Iran, alignment with these standards could guide transparent, ethical policies in workforce planning, diaspora involvement, and data-informed evaluations.

### Cross-national comparisons and global insights

Iran’s workforce migration patterns resemble those in other developing nations but differ in policy results due to sanctions and governance challenges. India’s Talent Return Program and China’s Thousand Talents Plan leverage financial incentives, advanced research facilities, and clear career paths to repatriate expatriates [111]. Iran’s HTP has reduced some regional disparities but grapples with financial instability, urban bias, and administrative burdens [10,11,31,34].

Nigeria’s migration; fueled by corruption, infrastructure deficits, and professional discontent; mirrors Iran’s. Yet, several African nations partnering with the WHO on planning and incentives have improved retention [86]. Iran’s sanctions impede similar collaborations, curtailing external aid and expertise.

Compared to the WHO Global Code of Practice [74], Iran’s strategies often omit rigorous evaluations of effectiveness, cost-efficiency, coverage, and sustainability. Coercive elements, like mandatory rural service, heighten dissatisfaction and emigration, as evidenced in qualitative themes (S3 Table) [9,33].

High-income countries, such as Canada, employ the Global Talent Stream to expedite visas and ensure ethical recruitment [108]. Iran could adapt such practices through structural reforms promoting transparency, merit-based progression, and equitable access. Adopting India’s diaspora model for circular migration and knowledge sharing would foster “brain circulation” over outright loss.

A participant observed: “Joint international projects could incentivize return” (P14, male academic, England). Amid sanctions, virtual platforms and networks offer feasible avenues for expatriate engagement and reverse migration.

## Discussion

The retention of healthcare professionals in Iran represents a multifaceted endeavour influenced by the country’s social, economic, and health policy developments. Over four decades, Iran has achieved significant milestones in cultivating and maintaining its healthcare workforce, while addressing persistent challenges.

Following the 1979 Islamic Revolution, the health system emphasized equity, primary care expansion, and medical training renewal. In the 1980s, despite constraints from the Iran-Iraq War, the government advanced rural services through the Behvarz program, which substantially improved national health indicators. These efforts established a foundation for workforce dedication, as professionals contributed to system resilience amid adversity [5]. Initial outflows occurred, with some seeking advanced training or secure environments abroad, yet domestic opportunities for specialization fostered retention among many [62].

The post-war reconstruction phase (1989–1997) featured gradual economic stabilization, with resources allocated to infrastructure restoration. Iran expanded medical education and local service coverage, enhancing professional satisfaction through increased access to training and roles [6]. Studies from this period indicate that retention stemmed more from professional development prospects than from external discontent [74,54], Factors such as equitable resource distribution and institutional support promoted stability, enabling self-sufficiency in clinical specialties [9].

In the late 1990s and early 2000s reform era, global mobility and opportunities in Organization for Economic Co-operation and Development (OECD) countries heightened the appeal of temporary international experience for Iranian physicians and nurses. Nevertheless, robust graduate production sustained workforce capacity, with many professionals returning to contribute to national health goals [6]. Recognition of achievements and career pathways encouraged ongoing commitment.

The 2010s introduced the HTP (HTP) by the Ministry of Health and Medical Education (MOHME), which expanded healthcare access and elevated job satisfaction through improved remuneration and service equity [10,11]. These advancements created opportunities for professional growth and reduced regional disparities, bolstering retention. Funding challenges and currency depreciation, however, affected long-term viability, prompting renewed focus on supportive policies [119].

The COVID-19 pandemic (2020–2022) imposed substantial demands on Iran’s health system, akin to global experiences. Professionals faced burnout and intense workloads, yet the response highlighted resilience, including rapid domestic production of personal protective equipment and vaccines [7,120]. This period underscored workforce dedication, as many sustained services under difficult conditions, reinforcing factors like institutional loyalty and peer support that aid retention [29].

Recent economic pressures, inflation, and limitations in research funding have influenced the retention of healthcare professionals, especially nurses and general practitioners. In recent years, a significant number of specialists have sought opportunities abroad, which has impacted workforce ratios and placed additional demands on the remaining staff compared to international benchmarks [76,121]. Policy responses, including financial incentives for underserved areas, flexible contracts, and agreements for circular migration and skill repatriation, aim to enhance stability [38,87,88]. These initiatives, alongside the HTP’s successes, demonstrate opportunities to leverage Iran’s strengths in medical education and primary care for sustained workforce engagement [6,11].

Overall, healthcare professional retention in Iran aligns with a developmental framework, emphasizing balanced labor market dynamics rather than unidirectional loss. The analysis draws on the WHO’s Health Labor Market Framework (HLMF) [2], which identifies imbalances and inefficiencies as influences on workforce stability. Economic factors, including recent inflation, have affected purchasing power. However, competitive domestic incentives and professional recognition continue to promote retention among healthcare professionals [122]. This meso-level institutional view connects macroeconomic conditions to individual motivations, with intersectional elements revealing that nurses and general practitioners; often from lower socioeconomic backgrounds; benefit from targeted supports like equitable pay [24,29]. Women, especially nurses, gain from reduced socio-cultural barriers, such as enhanced career mobility, which strengthens commitment [25,46]. Qualitative data, including a nurse’s observation, “My earnings in Iran hardly cover extra expenses” (P3, nurse, Canada), complement scoping review evidence on wage structures [27,63]. Underemployment relative to education levels contributes to stress and mental health concerns among professionals, underscoring the need for aligned opportunities [123]. Economic stability, adequate salaries, and research funding emerge as key retention enablers, consistent with studies highlighting wage differentials with countries like the United States, Canada, and Germany [85,111–114,124–129].

Governance elements; such as policy coherence and inclusivity; represent systemic features observed in other middle-income countries [130]. Merit-based recruitment and promotions, as noted by participants, build trust and sustain talent [112,113,131,132]. Fragmented leadership in higher education affects support, yet reforms emphasizing accountability and merit align with World Bank recommendations for recognition and career progression to retain skilled personnel [127]. These patterns resonate with research in developing nations, where investments in healthcare and research enhance stability [109,110]. Socio-political aspects, including social security and trust, influence retention; participants valued employment security and safe environments as anchors [3,133,64]. The HLMF illustrates how these factors align labor supply with demand through competitive conditions [2]. Professionals abroad valued improved work environments and salaries but faced adaptation challenges; however, domestic equivalents; such as supportive policies and cultural ties; outweigh such drawbacks for many, encouraging sustained engagement in Iran.

These insights integrate with study findings, highlighting how historical achievements amplify retention opportunities. The HTP’s gains in equity and access point to scalable strengths, while pandemic responses reveal inherent workforce resilience. Cross-national models, such as India’s diaspora networks and China’s flexible returns, suggest adaptations like virtual collaborations to overcome sanctions [111]. Longitudinal assessments would refine these approaches, aligning with global standards like the WHO Global Code [74]. Comprehensive strategies integrating economic supports, professional empowerment, and international ties would reinforce Iran’s health system, promoting equitable and enduring workforce retention in line with Sustainable Development Goals [134,135].

The retention of healthcare professionals in Iran embodies a dynamic process shaped by the nation’s social, economic, and health policy landscape. Women and younger professionals experience retention patterns influenced by socio-cultural and personal factors, including social trust and ethical considerations [25,29,46]. These variations align with the micro-level behavioral aspect of the framework, where attitudes and social norms mediate stability. A nurse’s observation, “ Working in environments where professionals feel undervalued can make it difficult to envision a long-term future “(P8, female nurse, USA). This aligns with evidence from the scoping review indicating that social and professional inequities during the COVID-19 pandemic influenced workforce morale and commitment [29]. Female health workers often encounter context-specific challenges related to workplace expectations and social norms, while practitioners in rural or underserved areas experience heavier workloads that may contribute to burnout [72,109,136]. Personal aspirations for self-actualization and improved living standards, reinforced by educational and cultural goals, gain strength when family or friends abroad offer support [9,54,55]. Individuals from higher socioeconomic strata, including elites with international exposure, demonstrate greater adaptability to domestic opportunities [133]. The research identifies educational and social elements; such as university admission systems, centralized resource allocation, and social trust; that shape retention. Participants from Iran emphasized credible bureaucratic processes as motivators, noting that struggles with inefficiency foster alienation, particularly among younger cohorts. The International Labor Organization (2023) affirms that social cohesion and institutional trust serve as vital factors for talent retention [3,118].

Beyond individual and professional dimensions, skilled health worker dynamics affect population health indicators, such as maternal and child mortality, primary care access, and progress toward health-related Sustainable Development Goals (SDGs) [65,66]. This corresponds to the macro-level of the framework, where aggregated individual actions yield systemic effects on health metrics and SDG advancement. The outflow of physicians, midwives, and nurses impacts health systems at three levels: reducing provider density in rural areas; elevating workloads for remaining staff and affecting care quality; and diminishing resilience to shocks like epidemics or disasters. Such dynamics erode clinical capacity and public trust, prompting delayed care-seeking and reliance on informal providers, which compromises equity [25]. Consequently, regional disparities in reproductive services, vaccinations, non-communicable disease management, and antenatal care widen, slowing reductions in maternal and child mortality. This highlights retention as a systemic imperative with implications for national health outcomes. Comparable evidence from the OECD (2023) and Shishehgar et al. (2015) indicates that shortages disrupt service delivery and exacerbate inequalities [126,133,137].

Although evidence links workforce dynamics to health outcomes, causal pathways remain complex and context-specific. A WHO report on health workforce and international mobility notes that effects intensify in settings with pre-existing shortages, where losing key professionals disrupts services like emergency obstetric care or vaccinations [138]. This study prioritizes retention drivers and professional consequences, while recognizing that broader systemic evaluations demand longitudinal investigation.

Retention patterns among Iranian healthcare professionals reveal interconnected influences. Departures can create perceptions that prompt further exits, perpetuating cycles. Addressing these requires educational policy reviews, power decentralization, and trust-building via transparent participation.

Compared to the WHO Health Labor Market Framework and Eastern Mediterranean Regional Strategies, Iran; like many other middle-income countries in the region; faces persistent challenges in workforce retention, equitable distribution, and governance. These issues mirror global patterns in low- and middle-income contexts, where professional dissatisfaction and uneven policy implementation often drive migration [35].

Over the past decade, Iran has introduced several health sector reforms aimed at improving service coverage, strengthening primary healthcare, and expanding medical education capacity. These initiatives have contributed to greater access in underserved areas but remain constrained by financial instability, inflation, and uneven resource allocation. Recent policy adjustments have increasingly emphasized transparent recruitment processes, performance-based incentives, and improved working environments to enhance professional satisfaction and institutional credibility [139].

Despite these efforts, participants in this study expressed concern about the effectiveness and perceived fairness of certain policies, particularly mandatory service requirements. As one physician noted, “Mandatory service made me more determined to leave” (P1, doctor, Germany). Nevertheless, such programs continue to play a crucial role in extending health service coverage to rural and remote regions [11,84].

The findings resonate with the WHO Health Labor Market Framework (HLMF) [2], stressing supply-demand-quality equilibrium. The WHO Eastern Mediterranean Regional Strategy (2017–2030) advocates governance, equity, and gender-sensitive policies, pertinent to Iran’s reforms [3]. Insights from India’s diaspora engagement and China’s remuneration strategies offer models for global collaboration [74,111]. Retention challenges manifest in underserved areas, where shortages strain delivery. Iran sustains a robust primary healthcare network and exceeds income-group peers in health education output [140]. Platforms for diaspora engagement, such as joint research, fellowships, and virtual networks, could harness expatriate expertise for capacity enhancement without full relocation. Approaches like China’s Thousand Talents Plan and India’s Knowledge Diaspora Program provide adaptable frameworks for knowledge exchange and reverse engagement [111,115].

The results endorse multisectoral strategies attuned to global practices and national needs. A policy blend emphasizing retention, engagement, and development transforms pressures into brain circulation and system fortification [141].

### Assessing current policies: Identifying weaknesses and missed opportunities

Over the past decades, Iran has implemented various measures to enhance retention of its healthcare workforce. These efforts have navigated historical, economic, and structural influences, yielding notable advancements alongside areas for improvement. During the Iran-Iraq War (1980–1988), nationwide free healthcare programs and job creation initiatives prioritized essential services despite resource redirection. This period established an extensive primary healthcare network and promoted equitable access across rural and urban settings, fostering professional commitment through shared national goals [5].

Subsequent modernization of health facilities and expansion of specialized services built on these foundations, improving service quality and professional opportunities. External economic pressures, including international sanctions, have constrained access to advanced technologies and collaborations, while inflation and currency fluctuations have impacted salary values and incentive sustainability. Nevertheless, these developments have strengthened workforce capacity, with ongoing investments in training enabling self-sufficiency in clinical specialties [6,9].

Retention strategies, such as monetary incentives, professional development programs, and compulsory service schemes, have produced varied outcomes. They have expanded coverage in underserved regions and supported career progression, aligning with international evidence that participatory, transparent, and well-funded approaches maximize effectiveness [135,142]. Factors like equitable remuneration and institutional support have sustained engagement, particularly in primary care, where professionals value contributions to national health equity [10].

Iran’s global scientific diaspora constitutes a strategic asset. Research indicates that Iranian professionals abroad attain high academic and professional levels, offering potential for transnational partnerships, knowledge transfer, and investments in domestic systems. The Migration Policy Institute reports that 51% of Iranian immigrants in the United States hold advanced degrees, with 75% of educators at leading universities [143]. Structured engagement via collaborative research, virtual networks, and return fellowships could promote brain circulation, bolstering local innovation without necessitating relocation [111,116].

Sustainability of reforms faces hurdles from high costs, administrative demands, and inter-institutional coordination [34]. Curriculum updates to match labor market needs have met occasional resistance from established structures [9], and governance fragmentation has occasionally diminished management efficiency [63]. Retention enablers, including merit-based advancement and supportive policies, counter these by enhancing morale and trust [2,3]].

A multisectoral framework addresses these elements effectively. Inter-agency coordination, stable financing, and adaptive governance would reinforce resilience. Through policy commitment and partnerships among government, academia, and international entities, Iran can advance retention, equitable distribution, and system durability, leveraging its primary care strengths and educational output [67].

Despite significant migration pressures, Iran has demonstrated measurable achievements in retaining segments of its healthcare workforce. Many professionals who chose to remain in Iran identified factors such as professional identity, social belonging, and opportunities for career advancement as central to their decision. Stable employment, family proximity, and cultural attachment were also frequently cited as protective motivators, consistent with findings across other middle-income countries [144,145]. Furthermore, expanded postgraduate education, improved gender equity in medical careers, and enhanced leadership participation for women have strengthened the sense of professional purpose and national contribution among younger cohorts.

Economic motivations remain mixed; while salary levels lag behind international benchmarks, targeted incentives and the prospect of professional recognition within domestic institutions partially offset financial disparities. The perceived impact of one’s work on public health outcomes; particularly within Iran’s primary healthcare network; emerged as a strong source of intrinsic motivation. Professionals emphasized that direct engagement with communities and opportunities to mentor students generated a sense of fulfillment and civic responsibility that counterbalanced the appeal of migration. Additionally, reforms prioritizing merit-based promotions, administrative transparency, and opportunities for participation in decision-making processes have improved trust in institutional structures [146]. Collectively, these elements illustrate that retention in Iran is not merely a function of economic calculus but also of moral commitment, identity, and professional inclusion. Sustaining these achievements requires continued investment in equitable governance, professional development, and participatory management to consolidate the gains achieved in recent years.

Recent official reports suggest a declining trend in the emigration intentions of medical professionals in Iran, reflecting the impact of a strategic three-pillar policy framework introduced by the Ministry of Health. According to Dr. Seyed Jalil Hosseini [146], Deputy Minister of Education, data from the Iranian Medical Council indicate a measurable reduction in the number of health professionals requesting documentation to support overseas migration. In official narratives, this shift is attributed to a strategic three-pillar policy framework introduced by the Ministry of Health, which emphasizes quality enhancement, equity, and administrative facilitation in medical education and workforce governance. These reports highlight measures such as efforts to ensure rigorous educational standards, more equitable treatment of students and faculty, and the simplification of selected bureaucratic processes without compromising academic or professional requirements. Strengthened communication channels between university leadership and academic staff, increased opportunities for participatory governance, and initiative to improve workplace satisfaction are also described as contributing factors. Some institutional sources further link these developments to recent appointments at the presidential and ministerial levels, which are portrayed as fostering a more supportive institutional climate. Concurrently, reports note a decline in certain self-reported indicators of psychological distress among medical students, suggesting broader well-being benefits from these systemic changes; however, systematic longitudinal evaluation would be needed to clarify the extent to which these trends can be attributed to recent policy changes. Despite persistent economic pressures; including international sanctions and relatively low levels of health financing; Iran’s academic and health sectors have shown notable resilience through sustained investment in training and service delivery. Recent, more targeted policy initiatives that respond to workforce concerns are reported to support professional retention and to attenuate some drivers of outward mobility, although systematic evaluation of their long-term effects is still lacking [146].

### Policy implications and retention strategies

Building upon the thematic findings, this section synthesizes evidence-based policy options to enhance the retention of the health workforce in Iran, while addressing structural and contextual factors that may influence migration intentions. The analysis draws on the WHO’s Global Code of Practice on the International Recruitment of Health Personnel [74] and aligns with national policy priorities outlined by the MoHME.

At the macro level, the results indicate that economic constraints, inflation, and global market inequalities remain important contextual influences on workforce stability. Addressing these issues through economic stabilization, equitable compensation mechanisms, and ethical international collaboration is essential for sustaining motivation among skilled professionals. Nevertheless, Iran’s health system continues to demonstrate resilience and adaptability through reforms designed to strengthen service delivery, integrate primary healthcare, and expand health coverage [35]. Continued investment in financing sustainability and local production of health technologies can further reinforce system stability and staff confidence.

At the meso level, institutional reforms within Iran’s healthcare infrastructure are vital for promoting organizational commitment and professional satisfaction. Key strategies include improving working conditions, enhancing managerial autonomy of hospitals, and establishing transparent, merit-based promotion pathways. The expansion of continuing professional development (CPD) programs and performance-based evaluation systems can improve job satisfaction and reduce turnover intentions [60]. Empirical evidence from the WHO Working for Health 2022–2030 Action Plan confirms that strengthening institutional credibility and leadership capacity supports long-term workforce retention [120].

At the micro level, individual and community-driven incentives are particularly influential. Mentorship programs for early-career physicians and nurses, psychosocial support for high-stress occupations, and recognition of professional achievements contribute to higher morale and stronger identification with the health system. Local recruitment from rural or underserved areas has been shown to enhance community engagement and retention through social embeddedness and trust [45]. As one participant noted, “Mandatory service made me more determined to leave” (P1, doctor, Germany), highlighting that while compulsory programs may extend rural coverage, retention improves most effectively when participation is perceived as fair, supportive, and rewarding.

More broadly, the Iranian health system possesses several structural advantages that can support workforce sustainability. These include an extensive network of primary healthcare centers, a strong medical education infrastructure, and growing research capacity supported by national initiatives [7]. Harnessing these assets through strategic governance, fair distribution of human resources, and evidence-informed workforce planning can strengthen professional identity and institutional trust; two core determinants of retention.

Finally, a multisectoral and forward-looking approach is recommended, integrating health policy with education, labor, and foreign affairs. Strengthened inter-ministerial coordination and the integration of workforce data systems will facilitate evidence-based decision-making and proactive management of retention risks [135]. These strategies align with Iran’s commitment to Sustainable Development Goal (SDG) 3.c, which emphasizes the development, training, and retention of the health workforce in all countries [147].

In conclusion, effective retention of health professionals in Iran requires concurrent actions at the macro, meso, and micro levels. By combining economic resilience, institutional reform, and individual empowerment, the country can sustain a motivated, equitable, and competent health workforce, enhancing both national health outcomes and the long-term resilience of its healthcare system.

### Proposed strategies: A systematic approach to retention and reverse migration

A systematic approach proves essential for bolstering healthcare workforce retention and encouraging reverse engagement in Iran. The following strategies draw on thematic evidence to promote stability and professional fulfillment.

#### Economic and professional incentives.

Enhancing economic stability and professional satisfaction remains a central priority [146]. Competitive remuneration structures, particularly in strategic sectors such as healthcare and education, can significantly improve retention. Salary schemes should be reviewed periodically to account for inflation, living costs, and labor market dynamics, while promoting equity across gender and regions [18]. Increasing investment in research, innovation, and advanced medical technologies would not only enhance service quality but also make the professional environment more attractive to skilled experts [34]. Establishing structured career development pathways; including mentorship, leadership training, and international exchange programs, can further support professional growth [50]. Comprehensive welfare policies, including health insurance, retirement benefits, and leave entitlements, may also boost job satisfaction and long-term commitment [10].

#### Governance and institutional reforms.

Enhancing governance mechanisms is pivotal for bolstering workforce retention. The implementation of transparent recruitment processes, meritocratic promotion systems, and standardized performance evaluation frameworks can contribute to increased institutional trust and employee motivation [67]. Streamlining administrative procedures associated with licensing, accreditation, and resource distribution may alleviate professional dissatisfaction [10]. Moreover, augmenting the autonomy of universities and healthcare institutions, especially in areas such as resource management and academic collaboration, has the potential to stimulate innovation and promote international engagement [27]. Additionally, establishing robust accountability mechanisms alongside inclusive decision-making structures can effectively address workforce concerns related to career progression and equitable treatment, thereby cultivating a more supportive professional environment [34].

#### Diaspora engagement and collaboration.

The scientific and professional diaspora of Iran constitutes a significant resource for the country’s development [12,140]. The creation of a specialized platform or a coordinating office dedicated to diaspora engagement could enhance communication, facilitate knowledge exchange, and promote investment opportunities [13]. Implementing flexible strategies; such as streamlined visa processes, short-term academic exchange programs, or visiting fellowship initiatives; may incentivize diaspora involvement in national projects [106]. Support mechanisms for returning professionals, including housing assistance, tax benefits, and integration into professional networks, could alleviate challenges associated with reintegration [113]. Furthermore, fostering collaborative research and teaching partnerships with expatriate scholars has the potential to transform the phenomenon of “brain drain” into “brain circulation,” thereby generating reciprocal advantages for both domestic and international academic institutions [111].

#### Social and cultural strategies.

Social recognition and cultural inclusion constitute equally vital elements in the retention of professionals [34]. Incentives grounded in recognition; such as national honors, professional designations, and media exposure; serve to enhance motivation and reinforce a sense of professional pride [102]. The promotion of cultural initiatives that honor both Iran’s historical heritage and its contemporary accomplishments may cultivate a heightened sense of belonging and national identity [117]. Additionally, the establishment of professional associations and peer networks can foster solidarity and mitigate professional isolation [144]. Furthermore, broadening access to mental health and counseling services for healthcare practitioners is essential in addressing psychological stressors, including burnout, loneliness, and dissatisfaction, which frequently act as antecedents to migration [98,136].

#### International collaboration and integration.

Enhancing international engagement continues to be a fundamental strategy in managing workforce migration [15]. Increasing involvement in global health, education, and research collaborations can improve professional exchanges and elevate institutional prominence [120]. Establishing cooperative frameworks with esteemed academic and medical institutions may enable joint research initiatives, faculty exchanges, and student mobility programs [113]. Moreover, active participation in international organizations, including the WHO and the United Nations, offers critical access to capacity-building resources and technical support [117]. Additionally, cultivating an environment conducive to international collaboration and investment; especially within the healthcare and education sectors; has the potential to create employment opportunities, foster innovation, and enhance the sustainability of systems [127].

#### Educational reforms.

Harmonizing educational curricula with current international standards and technological advancements is essential to prepare graduates effectively for the complexities of contemporary healthcare. Strengthening vocational and professional training programs, especially in fields such as nursing, laboratory sciences, and health administration, can mitigate skill deficits in underserved regions. The expansion of scholarship initiatives, research fellowships, and international exchange programs has the potential to motivate students to engage in advanced studies and subsequently apply their expertise toward national development objectives. Moreover, revising curricula to incorporate competencies in digital health, healthcare management, and global health will better align educational outcomes with the dynamic requirements of the healthcare workforce [146].

#### Technological innovation.

The utilization of digital health technologies has the potential to enhance healthcare delivery and alleviate pressures on the healthcare workforce [129]. Implementing telemedicine, electronic health records, and artificial intelligence applications can improve operational efficiency and expand access to medical services in underserved and remote regions [2]. Furthermore, encouraging hybrid and remote work arrangements; especially for activities such as consultations, continuing professional education, and administrative functions; may help address factors contributing to workforce migration by fostering a better work-life balance [136]. Additionally, the establishment of comprehensive data systems to track migration patterns and workforce distribution is essential for facilitating evidence-based policy development and enabling prompt, informed interventions [31].

#### Environmental and quality of life improvements.

Investments in both urban and rural environments that promote livability play a significant role in enhancing overall workforce satisfaction [87]. Increasing the availability of affordable housing for healthcare professionals, especially in metropolitan and underserved areas, can mitigate financial burdens [112]. Additionally, advancements in public transportation, workplace safety, and environmental health standards are essential for improving quality of life and decreasing occupational burnout. The incorporation of these strategies into comprehensive social welfare and urban development policies is likely to foster greater retention and commitment among healthcare professionals [131].

#### Decentralized and regionally tailored strategies.

Retention dynamics exhibit considerable variation among the provinces of Iran, indicating the necessity for policies tailored to specific regional contexts [100]. Implementing decentralized strategies that grant autonomy to regional medical universities and health authorities to develop retention initiatives adapted to local conditions may enhance both the responsiveness and sustainability of such policies [147]. Regionally targeted incentives; including housing programs, localized career development opportunities, and selective salary modifications; have the potential to more effectively address the distinct factors influencing migration [34]. Furthermore, the establishment of regional health workforce councils could facilitate improved policy coordination and foster greater local ownership [147].

#### Professional autonomy and participation in decision-making.

Enabling healthcare professionals to engage actively in institutional and policy-level decision-making processes has the potential to improve job satisfaction and decrease turnover rates [102]. Empirical evidence indicates that participation in the development of clinical guidelines, health policies, and administrative procedures cultivates a sense of ownership and reinforces professional identity. Promoting inclusive governance structures that incorporate the viewpoints of physicians, nurses, and researchers may enhance morale, foster a positive organizational culture, and support workforce retention [147].

#### Flexible return pathways and circular migration models.

Developing adaptable return strategies for diaspora professionals represents a novel method to counteract brain drain. Instead of mandating permanent resettlement, facilitating short-term visits, remote collaboration, and joint appointments can foster ongoing involvement with home-country institutions [111]. Circular migration frameworks permit overseas professionals to contribute via teaching, research, and consultancy activities, thereby enabling the exchange of knowledge and expertise without necessitating a full-time return [125]. The establishment of institutional agreements and incentive structures to support these mechanisms has the potential to convert migration into a mutually beneficial process that enhances capacity building [109].

#### Peer-based retention and professional community building.

Enhancing peer-driven professional communities constitutes a relatively underexploited but highly efficacious strategy for cultivating mutual support networks among healthcare practitioners [144]. Engagement in professional networks at both national and international levels has been demonstrated to bolster professional identity, alleviate feelings of isolation, and promote collaborative practice. These networks have the potential to mitigate burnout and emotional exhaustion, which are significant determinants influencing healthcare workers’ decisions to migrate [71,136]. Consequently, the development of mentorship programs, professional associations, and interdisciplinary collaborations may serve to reinforce solidarity and enhance the sustainability and resilience of the healthcare workforce over time [102].

### Evaluation of proposed strategies

S7 Table provides a comprehensive evaluation of the proposed strategies based on their effectiveness, sustainability, and feasibility. Notably, decentralized governance models, enhancement of professional autonomy, circular migration frameworks, and economic-professional incentives emerged as the most promising and contextually appropriate interventions for Iran’s healthcare system. Economic reforms exhibited considerable effectiveness by targeting fundamental structural factors driving migration, such as insufficient remuneration and limited opportunities for career progression [20,109]. Although fiscal limitations pose challenges to implementation, the long-term sustainability of these reforms can be attained through phased financial planning and the strategic prioritization of essential workforce segments. Governance reforms were deemed crucial for promoting equity and transparency within the system [147]. Despite encountering administrative and political obstacles, these reforms are regarded as highly sustainable once they become institutionalized. Socio-cultural and diaspora engagement strategies demonstrate moderate effectiveness but exhibit considerable sustainability, attributable to their capacity to cultivate enduring professional networks and reinforce identity [79]. International collaboration presents substantial potential for enhancing professional exchange and capacity building [15]; however, its influence is somewhat limited by external contextual variables. Educational reforms, when aligned with international standards and complemented by ongoing professional development, consistently achieve both high effectiveness and sustainability. Likewise, advancements in technology and improvements in quality of life can significantly improve workforce retention by mitigating occupational stressors and extraneous push factors, though their full benefits necessitate coordinated, multisectoral investment. The integration of economic–vocational reforms with diaspora engagement and educational modernization proves to be both pragmatic and impactful. Reforms in governance and international collaboration exhibit strong potential effectiveness but may require phased implementation due to inherent systemic complexities. Given the semi-autonomous nature of Iran’s regional medical universities, decentralized retention strategies are particularly feasible, as they allow for tailored interventions responsive to local conditions while maintaining alignment with national objectives [31]. Models of professional autonomy and participatory governance are viable options, as they can be effectively implemented through existing professional associations and health councils. Empowering healthcare professionals to engage in decision-making processes enhances their motivation, decreases attrition rates, and promotes accountability [102]. Circular migration frameworks, which allow for short-term involvement or remote collaboration by Iranian professionals working abroad, exhibit considerable feasibility and long-term sustainability [111]. These frameworks support continuous professional exchange, encourage reciprocal learning, and mitigate the emotional and logistical challenges associated with permanent repatriation. Furthermore, community-based and peer-support initiatives represent a cost-effective and impactful approach. Reinforcing these professional networks can cultivate a lasting sense of belonging and collective purpose, thereby contributing to the stability and resilience of the workforce over time [121].

The choice of strategies must take into account the resources at hand, the capacity of institutions, and the feasibility within a medium-term timeframe. Together, these interventions have the potential to address the economic, social, and institutional determinants driving the migration of healthcare personnel. In the absence of a systematic response, the ongoing depletion of the workforce is likely to persist, thereby exacerbating pressures on the national health system and heightening dependence on external recruitment [125]. In contrast, a coordinated, evidence-informed, and inclusive strategy can alleviate negative consequences and enhance the sustainability of the health system.

### Study limitations and future directions

This study presents several limitations. The sample size within specific subgroups, such as female physicians and nurses practicing in rural areas, was relatively small, potentially compromising the representativeness of the findings. Although the scoping review included gray literature, certain institutional reports that have not been digitized may have been omitted. Furthermore, the lack of qualitative analysis software constrained the automation of coding processes; nonetheless, manual thematic coding enabled a more nuanced and in-depth interpretive engagement with the data. Future research endeavors should consider employing mixed-methods or quantitative approaches to validate the emergent themes and to evaluate their prevalence on a national scale. Comparative studies involving neighboring countries, such as Iraq or Jordan, could provide valuable contextualization of Iran’s experiences within the broader regional framework. Additionally, focused investigations targeting underrepresented populations; such as early-career healthcare professionals and female nurse migrants; would contribute to a more comprehensive, intersectional understanding of the factors influencing migration. Finally, the incorporation of the WHO’s Health Labor Market Framework into Iran’s policy formulation and monitoring mechanisms could enhance the coherence between national and regional health workforce strategies. The development of evidence-based, equity-focused policies tailored to the specific local context will be essential for transforming the current brain drain phenomenon into a sustainable cycle of professional development and national advancement.

## Conclusion

This study underscores that health workforce retention in Iran demands strategic and sustained policy attention, as it is shaped by a confluence of economic, professional, and structural factors. While international mobility remains a reality, the evidence suggests that Iran possesses significant opportunities to mitigate its adverse effects and harness existing strengths within its health system. The country’s robust medical education infrastructure, extensive primary healthcare network, and recent policy innovations; such as elements of the HTP; demonstrate a foundational capacity for effective retention strategies.

Our integrated analysis of qualitative insights and scoping evidence reveals that meaningful improvements in retention are achievable through coherent, system-wide reforms. Key enablers include equitable remuneration, clear career progression pathways, supportive work environments, and institutional trust. Notably, retention is not solely contingent on preventing outward mobility but on fostering enduring professional engagement, whether individuals reside domestically or abroad. In an interconnected global landscape, the critical factor is not physical presence alone, but the sustained connection of health professionals to national development through knowledge exchange, mentorship, and collaborative innovation.

Iran has the potential to reframe the challenge of workforce mobility from a deficit narrative toward a strategic opportunity, transforming potential loss into a dynamic form of brain circulation. Realizing this vision requires moving beyond reactive measures toward long-term investments in governance reform, sustainable health financing, and structured international engagement mechanisms that facilitate reciprocal collaboration. Future research needs to assess the practical effects of proposed interventions on underrepresented groups and regions while developing scenario-based foresight models to help policy makers adapt to fast-changing global health conditions.

## Supporting information

S1 Table Summary of extracted data from empirical articles. (DOCX)

S2 Table Summary of extracted data from policy and institutional documents. (DOCX)

S3 Table Themes, subthemes, and supporting evidence on health workforce retention in Iran. (DOCX)

S4 Table Annual publication counts on the retention of Iranian health personnel. (DOCX)

S5 Table Frequency of retention factors reported in the articles. (DOCX)

S6 Table Comparative evaluation of health workforce retention policies in Iran across four key dimensions: effectiveness, coverage, cost-efficiency, and sustainability. (DOCX)

S7 Table Assessment of retention and reverse migration strategies in Iran based on feasibility, effectiveness, and sustainability. (DOCX)

S1 Appendix Semi-structured interview guide. (DOCX)
