# Supplementary material for: Determinants and policy approaches to healthcare professional retention in Iran: A mix of scoping review and qualitative evidence
Source: PLoS One. 2026 Apr 21;21(4):e0339855. doi: 10.1371/journal.pone.0339855 (PMC13099093; doi:10.1371/journal.pone.0339855)
Supplement: S1 Table — (DOCX) [file pone.0339855.s001.docx]

**Table 1:** Summary of Extracted Data from Empirical Articles

| No. | Authors & Year | Study Design | Study aim | Target Group | Reasons for Migration | Recommendations | Possible Effects | Benefits of the recommendation | Disadvantages |
| --- | --- | --- | --- | --- | --- | --- | --- | --- | --- |
| 1 | V. Shojaeimotlagh, 2018 (27) | Qualitative | Exploring the psychological factors motivating Iranian nurses to migrate. | Nurses | Lack of job satisfaction, authority, social support | Address psychological needs, improve mental health, job promotion opportunities | Higher job retention, reduced migration | Improved well-being | Sustained support needed |
| 2 | H. Asadi, B. Ahmadi, 2016(19) | Cross-sectional | Identify factors influencing migration intentions. | Health workers | Better life opportunities, discrimination, prior experience abroad | Develop targeted policies, assess immigration status, analyze social and economic factors | Enhanced retention strategies | Improved workforce planning | High implementation costs |
| 3 | Asadi et al., 2018 (9) | Qualitative | Assess determinants of specialist migration in Iran's healthcare. | Healthcare specialists | Structural, occupational, socio-political, and economic issues | Improve job security, revise workforce curricula, address income distribution | Better workforce stability | Equitable income distribution | Policy adjustments required |
| 4 | HA Ronaghy, A Shajari, 2013(94) | Descriptive Analytical Study | Gather data on Iranian medical school graduates. | Physicians | Push factors in Iran, pull factors in the US | Restrict migration, develop postgraduate programs in Iran | Retain medical graduates | Increased local expertise | Restrictive measures |
| 5 | F. Shams, A. Shams, 2014(72) | Letter to the Editor | Challenges of emigration. | Medical students | Poor postgraduate prospects, lack of referral systems | Enhance education quality, improve income distribution | Better postgraduate retention | Higher career satisfaction | Challenging implementation |
| 6 | E.C. Elham et al., 2018(52) | Qualitative | Identify key factors for physician retention | Public-sector physicians | Lack of infrastructure in underdeveloped provinces | Develop infrastructure, promote equity across provinces | Improved workforce distribution | Enhanced service access | High resource demands |
| 7 | M. Fardid et al., 2018(26) | Cross-sectional | Evaluate links between organizational justice, commitment, job satisfaction, and turnover intention. | Nursing staff | Organizational injustice, low commitment | Promote procedural justice, equitable reward systems, transparency | Improved retention, satisfaction | Fairer workplaces | Complex implementation |
| 8 | B. Damari, E. Ehsani-Chimeh, 2019(86) | Mixed-method | Examine HRH management status in Iran | Human resources managers and experts in the health sector | Management gaps in HRH | Form committees, prioritize HRH policy execution | Strategic HRH planning | Sustainable workforce planning | Requires long-term commitment |
| 9 | S. Vatankhah et al., 2019(73) | Systematic review | Investigate factors contributing to nurses’ outflow from Iran’s healthcare system. | Nurses | Cultural, economic, and organizational factors | Improve staffing, foster positive culture, flexible work plans | Higher participation, retention | Better teamwork | Requires systemic changes |
| 10 | Z. Nikkhah-Farkhani et al., 2020(55) | Descriptive cross-sectional | Compare factors affecting nurses’ turnover between Iran and Poland | Nurses | Work-family conflict, lack of workplace support | Flexible work plans, better cooperation in workplaces | Higher job satisfaction | Improved cooperation | Limited scalability |
| 11 | N. Eissazade, D. Hemmati, 2021(25) | cross-sectional study | Investigate the extent and motivations of migration among psychiatric trainees | Psychiatrists | Professional, financial, and academic challenges | Enhance political stability, improve work conditions and finances | Increased retention | Greater workforce equity | Resource-intensive reforms |
| 12 | H. Zahednezhad et al., 2021(28) | Cross-sectional | Test a model connecting organizational justice to job satisfaction and nurses' turnover intentions | Nurses | Organizational justice gaps | Implement distributive and interactional justice mechanisms | Reduced turnover intention | Higher satisfaction | Complex integration |
| 13 | S. Varasteh et al., 2022(29) | Cross-sectional | Examine factors influencing nurses' decisions to leave or remain in their profession | Nurses | Emotional exhaustion, reduced accomplishment | Support during crises, promote retention measures | Workforce stabilization | Enhanced resilience | Time-sensitive support |
| 14 | S. Hajian et al., 2023(16) | Qualitative | Explore migration motivations of Iranian dentists to Canada | Dentists | Limited professional autonomy and social constraints affecting job satisfaction | Respect for individual rights, enhance social security | Improved retention of dentists | Better quality of life | Cultural adaptation challenges |
| 15 | M.Taherahmadi et al., 2023(24) | Cross-sectional study | Investigate factors affecting migration among medical students | Medical students | Higher socioeconomic status, previous exposure to foreign countries | Enhance academic opportunities, improve language skills | Increased migration readiness | Potential loss of local talent | Limited resources for implementation |
| 16 | AA. Haghdoost & M. Fasihi Harandi, 2022(34) | viewpoint | Address challenges for academic staff retention | Academic staff | Challenges in the private sector include financial strain, slow retirements, heavy workloads, limited academic opportunities, job burnout, poor facilities, and low decision-making involvement. | Reform pension laws, improve university management | Enhanced job satisfaction | Resistance to change | Bureaucratic delays |
| 17 | Am. Mosadeghrad & F. Moghadasi, 2023(46) | Letter to the editor | Investigate causes and consequences of nurse migration | Nurses | Economic instability, poor working conditions | Implement training and retention measures | Improved working conditions | Increased retention | Limited resources for implementation |
| 18 | R. Pourafshar et al., 2022(87) | Qualitative interviews | Provide solutions to prevent migration crisis | Health elites | Lack of opportunities, better quality of life abroad | Improve governance, enhance job recognition | Retained elite talent | Potential for bureaucratic delays | Implementation complexity |
| 19 | A. Jafarian & A. Parsapour, 2022(83) | Opinion, | Analyze migration trends among medical professionals | Medical professionals | Perceived inequities in career advancement opportunities | Foster economic growth and job creation | Improved healthcare quality | Economic constraints | High inflation impact |
| 20 | S. Rahmati et al., 2024(74) | Qualitative study | Investigate migration reasons for epidemiologists | Epidemiologists | Insufficient income, better job opportunities abroad | Enhance educational and professional status | Increased motivation to stay | Resource allocation challenges | Limited funding |
| 21 | A. Ghaffarian & M. Ferdosi, 2023(81) | Survey study | Examine willingness of doctors to immigrate | Doctors | Economic instability and resource limitations, inadequate facilities | Improve salary and working conditions | Better retention rates | High inflation impact | Limited government support |
| 22 | Z. Yazdani et al., 2024(31) | Qualitative | Design specialty and subspecialty supply model | Health workforce | Limited international scientific engagement and employment-related challenges | Create a customized planning model | Optimized workforce management | Enhanced job satisfaction | Implementation complexity |
| 23 | AA. Haghdoost, S. Nouri Hekmat 2022 (18) | Descriptive | Investigating factors affecting human resource migration | Health Workforce | Economic factors, management, social welfare | • Serious commitment to elite management  • Structural and legislative reforms  • Meritocracy  • Increasing hope in academic community | - Increased hope in the academic community | -Improved management and retention of health workforce.  - Long-term commitment required | - Complexity in implementation |
| 24 | M. Jannesar, SM. Seyedi, 2023 (123) | Mixed-Method Study | Investigating migration drivers | Health Professionals | Non-futuristic expansion of university capacity without quality consideration | • Revision in education, research, and technology  • Allocate 4% of GDP to research  • Strengthen top scientific centers  • Address immigration roots  • Meritocracy in elections  • Strengthen patriotism | - Enhanced research quality and relevance | - Potential for improved educational outcomes | - Resistance to change in established systems |
| 25 | S. Nouri Hekmat, M. Maleki 2010(95) | Cross-sectional study | Identify factors encouraging graduates to migrate | Medical students | Economic, administrative, educational, professional, globalization, social and cultural factors | • Align university courses with societal needs  • Enhance student skills  • Strengthen industry-university ties  • Expand educational and job opportunities | - Improved student skills | - Better alignment with job market needs | - Potential resistance from traditional educational structures |
| 26 | F. Nazari Rabati, M. Sadeghi (2023)(93) | Field Review Study | Investigating strategies to strengthen brain circulation and attract elites | Health Elites | Push and pull factors | • Governance reforms  • University reforms  • Cultural strategies  • Promote remote services  • Support economic participation and development projects | - Enhanced circulation of health elites | - Improved health system efficiency | - Implementation challenges due to policy changes |
| 27 | F. Aladdini, R. Fatemi (2005)(124) | Survey Study | Examining doctors’ willingness to immigrate | Doctors | Economic pressures, inflation, and perceived inequities in workplace conditions, need for career advancement, lack of amenities | Fundamental changes in health system management structure | - Increased willingness to stay | - Improved working conditions | - Resistance to structural changes |
| 28 | H. Akbari Javar, A. Khalaj (2018)(84) | Descriptive survey | Investigating migration status of pharmacy graduates | Pharmacists | Continuing education, better job position, higher income, well-being facilities | - Enhance pharmacists' roles by defining new positions  -Improve training and facilities  -Involve pharmacists in semi-medical decisions  – Foster a positive public perception | - Improved job satisfaction and retention | - Better alignment with healthcare needs | - Potential resistance from traditional roles |
| 29 | S. Kazemi (2022)(137) | Qualitative Study | Explain the phenomenon of doctor migration based on the views and experiences of medical interns | Internship medical students | Medical education status, job conditions, declining social status of doctors, poor work-life balance, demotivation, external influences | - Review medical education system  -Improve living and working conditions of doctors  -Transfer medical education oversight to the Ministry of Science  -Modify teacher-student relationships  - Increase intern salaries  -Standardize working hours  -Set up a referral system  -Ensure professor accountability | - Enhanced working conditions for doctors | - Improved morale and retention | - Potential pushback from existing educational structures |
| 30 | M Motie, N Arsalani (2024)(138) | Narrative review | Reviewing factors contributing to global nurse migration | Nurses | Psycho-social, organizational, economic and individual-family factors | Detailed analysis of causes, effects and solutions related to immigration | - Improved understanding of migration dynamics | - Better targeted interventions | - Potential for limited applicability of findings |
| 31 | M. Dastjerdi & N. Sultankhah, 2021(132) | Qualitative | Identification of challenges and priority strategies for health elites | Health Elites | 27 challenges including reduced medical field entry quotas, inadequate higher education support, lack of execution guarantees for elite-related tasks | • Identify and nurture elites early in life  • Preserve country’s independence and progress through meritocracy  • Respect and effective resource utilization | - Enhanced retention of health elites | - Improved national health outcomes | - Challenges in implementation across various sectors |
| 32 | H. Derakhshan Shahrabad, 2023(139) | Review | Reviewing causes of emigration of medical and non-medical Iranian elites | Medical and Non-medical Elites | Economic, structural, occupational, personal, and socio-political factors | • Prioritize economic factors  • Ensure education continuity  • Recognize expertise  • Strengthen university-industry ties  • Enhance local employment opportunities | - Improved local employment opportunities | - Potential resistance to change from established institutions | N/A |
| 33 | M. Tajari & T. Ashktorab, 2023(140) | Qualitative | Identify the reasons behind Iranian nurses’ immigration | Nurses | Professional disappointment, lack of professional value, social chaos, economic chaos, promotion issues | - Provide a suitable environment for employment and education with reasonable salary and facilities | - Improved retention of nurses | - Enhanced working conditions | - Potential implementation challenges |
| 34 | M. Ghanbari-Jahromi Ma. Marzaleh 2024(75) | Qualitative | Identify factors affecting brain drain and solutions to reduce it in Iran’s health system | Iranian health experts and emigrant elites | Individual, economic, social, cultural, organizational, administrative, Governance and administrative challenges, attraction factors for destination countries | - Major systemic changes to improve work conditions | - Increased workforce stability | - Long-term commitment required | - Resistance to systemic changes |
| 35 | M. Okhovati et al., 2023(119) | Multi-centric cross-sectional | Investigate inclination to migrate among medical residents | Medical residents | Disproportionate income, better welfare in destination countries, Employment-related challenges and limited professional incentives | - Improve educational and professional status of residency programs by adjusting shift hours and salaries | - Increased motivation to enter residency | - Better alignment with workforce needs | - Potential resistance from existing programs |
| 36 | S. Vakili & M. Mobini, 2023(76) | Review, | Overview of brain drain causes and policy issues in Iran | General population | Push-pull factors, higher income, attractions of destination countries, individual and family factors | - Investigate and manage brain drain using established theories | - Improved understanding of migration dynamics | - Better-informed policy decisions | - Complexity in addressing multifaceted issues |
| 37 | M. Zamanian & N. Khangolzade, 2024(77) | Research paper | Examine evolution of migration governance in Iran | Elites | Strengthening national image and improving communication strategies regarding workforce mobility, redefining immigration governance | - Create capacities for attraction and growth of international private companies | - Enhanced national image | - Potential for improved investment | - Challenges in policy implementation |
| 38 | A. Mehdi, 2020(82) | Qualitative | Analyze characteristics of youth emigration from Iran | Young professionals | Economic constraints, Economic and professional constraints, and differential employment conditions | - Economic initiatives and job creation | - Retained national talent | - Long-term commitment required | - Potential resistance from established norms |
| 39 | B. Movahedi et al., 2024(60) | Qualitative | Analyze the institutional environment of migration management | Policy makers, government institutions | Not specified | • Implement governance reforms  • Focus on immigration policies  • Conduct university reforms  • Develop cultural strategies  • Promote remote services  • Support economic participation | Creation of a national migration management discourse | Comprehensive approach to migration management | Not reported |
| 40 | S. Biglari et al., 2023(69) | Mixed research approach | Investigate future behaviors of Iran’s brain drain and explore effectiveness of policy interventions | Skilled individuals in Iran | • Economic challenges  • Limited career opportunities  • Social and institutional factors | • Focus on human capital efficiency  • Implement systemic policy interventions  • Address underlying cultural and societal issues • Improve economic conditions  • Enhance career development opportunities  • Address social and political concerns | -Potential reduction in brain drain over time  • Increased retention of skilled individuals  • Improved national development | • Comprehensive analysis of the issue  • Integration of multiple perspectives  • Long-term policy planning • Holistic approach to a complex issue  • Potential for long-term positive impact | • Complexity of implementation  • Requires significant resources and time • May require significant systemic changes  • Results may take time to materialize |
| 41 | Z. Yazdani, P. Saketi, F. Karimi, M. Yarmohammadian (2018)(70) | Mixed-method study | Identify factors affecting retention of native Iranian physicians and preventing migration abroad | Native Iranian physicians | - Better learning opportunities abroad - Low quality of education - Lack of updated medical facilities and training - Limited research opportunities - Economic challenges and contextual limitations in international collaboration | - Establish strong educational management - Flexible work schedules with part-time contracts - Improve research opportunities - Equip laboratories with advanced technology - Encourage teamwork and knowledge sharing - Enhance international interactions - Promote culture-building for anatomical resources and training - Develop virtual learning platforms and updated educational materials - Ensure equitable distribution of patients - Address economic and gender disparities | - Increased retention of physicians - Improved health services - Reduction in physician migration - Strengthened national medical research and innovation | - Enhanced education and training systems - Better work-life balance for physicians - Improved research and technological advancements in the medical field | - Economic and cultural barriers to implementation - Potential resistance to policy changes - Challenges in securing funding for proposed solutions |
| 42 | Z.Arabbadvi,(2025),(38) | Scoping Review | To understand the reasons for and approaches to managing health workforce migration in Iran | Iranian health workforce (all professions, national level) | - Governance (policy, management, resource allocation)  - Social (economic, job security, quality of life)  - Social scope (cultural, societal expectations) | - Governance/structural reforms  - Academic/educational improvements  - Individual/social support strategies | - Reduced workforce  - Lower healthcare access and quality  - Brain drain  - Systemic instability | - Improved retention  - Sustainable workforce  - Enhanced system resilience  - Better healthcare outcomes | - Requires multi-level coordination  - May need substantial resources  - Implementation challenges |
| 43 | M.Bahrami et al., (2025)(36) | Qualitative Content Analysis | To explore challenges Iranian nurses face when emigrating | Iranian nurses | Professional dissatisfaction, Economic and social challenges, and limited professional development opportunities, limited career advancement, cultural disparities | Improve economic conditions, enhance dignity and respect, address professional and social challenges | Strong emigration tendency, loss of skilled nurses, cultural and emotional challenges post-migration | Better nurse retention, improved job satisfaction, reduced emigration | Language barriers, admission complexity, homesickness, cultural adaptation difficulties |
