# Supplementary material for: Determinants and policy approaches to healthcare professional retention in Iran: A mix of scoping review and qualitative evidence
Source: PLoS One. 2026 Apr 21;21(4):e0339855. doi: 10.1371/journal.pone.0339855 (PMC13099093; doi:10.1371/journal.pone.0339855)
Supplement: S2 Table — (DOCX) [file pone.0339855.s002.docx]

**Table 2:** Summary of Extracted Data from Policy and Institutional Documents

| Challenges | Possible Effects | Intervention | Root Causes | Target Group | Goal | Issuer | Type | Author | Document Name/Title | No. |
| --- | --- | --- | --- | --- | --- | --- | --- | --- | --- | --- |
| Inefficient implementation and limited resources | Reduced inequality and prevention of forced migration/ Increased social justice, Universal rights enshrined | Free health services and job creation | Not reported | General population and workforce | Guarantee health and employment rights | Government of the Islamic Republic of Iran | National | Islamic Consultative Assembly | The Constitution of the Islamic Republic of Iran (Articles 29 and 43)(141) | 1 |
| lack of detailed operational plans | Increased regional competitiveness/ Focus on macro-level goals | Develop health infrastructure and attract experts | Economic and social factors, lack of job satisfaction | Specialists and health workforce | Achieve top regional health ranking | Government, Ministry of Health | National | Expediency Discernment Council | Iran’s 20-Year Vision Document (1404)(142) | 2 |
| Partial implementation in some regions, High financial costs | Reduced migration inclination/ Increased satisfaction | Increase wages and improve working conditions | Economic instability, lack of job opportunities | Healthcare personnel and specialists | Improve the health system and retain workforce | Ministry of Health and Planning and Budget Organization | National | Supreme Leader | General Policies of the Sixth Development Plan (Health Provisions)(78) | 3 |
| May not yield immediate results in the short term | Strengthened job satisfaction and elite retention, More trained physicians in 10–15 years | Draft supportive laws for specialists, Increase enrollment in medical schools, better healthcare distribution | Overemphasis on quantitative growth, declining education quality, lack of future opportunities | Students, healthcare workers | Expanding medical training capacity to meet future demands | Ministry of Health, Parliament | National | Parliament | Healthcare Policies in the Seventh Development Program (Proposed)(80) | 4 |
| May neglect other sectors | Enhanced motivation to stay in the country/ Focus on a specific group | Provide special facilities and financial support | Economic and social factors, lack of opportunities | Academic and health elites | Prevent brain drain | National Elite Foundation, Ministry of Health | National | Supreme Leader | Supreme Leader’s General Policies on Elites(79) | 5 |
| need for systemic reforms and sufficient funding | better job security, improve working conditions, and provide incentives to retain healthcare professionals​ | programs like "Physician Researcher" and the establishment of the NIMAD (National Institute for Medical and Health Development) to help retain medical talent/ timely recruitment and the provision of adequate employment opportunities for graduates/ Promotion of Health Tourism/ Supportive Policies and Systemic Reforms | Economic and contextual challenges affecting workforce stability | Health professionals in Iran | Reduce emigration of health professionals | Government of Iran | National | Ministry of Health | Healthcare Workforce Retention Plan(105)(106) | 6 |
| High Costs, Bureaucratic Challenges, Potential Inequity, Dependency on Government Support, Limited Effectiveness | Reduced brain drain, increased innovation | Comprehensive incentive packages (employment, education, benefits, military service solutions) | Lack of suitable employment, educational opportunities, benefits, and military service issues | Iranian elites, both domestic and abroad | Manage and control elite migration, create favorable conditions for return | Ministry of Science, National Elite Foundation, Supreme Council of Iranian Affairs Abroad | National | Leadership Consultant | Expediency Discernment Council(50) | 7 |
| Dependence on external factors, funding and infrastructure issues, and limited long-term elite engagement | Enhanced innovation, reduced brain drains, improved collaboration, and better alignment with national priorities. Strengthened global networks and problem-solving capacity | Recognize elite talent, expand research opportunities, provide scholarships and support, and create incentives for returnees. | Overseas education and opportunities, better conditions abroad, and domestic resource disparities. | Elite individuals and groups in science, arts, technology, humanities, and other fields of national significance. | Activate elite potential to achieve national objectives by 1404. | National Elite Foundation | National | Supreme Council of the Cultural Revolution | Strategic Document on National Elite Affairsaffairs(88) | 8 |
| High costs, potential for inefficiency | Improved accessibility and quality of healthcare / Long-term sustainability, Nationwide reforms to improve access | Healthcare system reforms | Economic and social factors, lack of job satisfaction | General population | Achieve universal health coverage | Ministry of Health | National | Iranian Ministry of Health | Health Transformation Plan (HTP) in Iran(10) | 9 |
| Requires international cooperation | Reduced harm to source countries/ Ethical compliance | Standardize and prevent unethical recruitment | Economic and social factors, lack of job opportunities | Global health workforce | Regulate the recruitment of specialists | World Health Organization (WHO) | International | WHO | Global Code of Practice on the International Recruitment of Health Personnel(58) | 10 |
| Difficult to enforce at national levels | Improved management of skilled migration flows/ Multilateral coordination | Establish global migration policies | Economic and social factors, Institutional and contextual challenges | Migrant workforce | Manage migration effectively | IOM | International | International Organization for Migration (IOM) | Global Compact for Migration(59) | 11 |
| Limited health sector-specific focus | Enhanced global economic stability/ Holistic migration insights | Economic policies for equitable migration | Economic and social factors, lack of job opportunities | Global economy | Assess economic impacts of migration | IMF | International | International Monetary Fund (IMF) | Migration and Economic Impact Reports(101) | 12 |
| High implementation costs and need for systemic changes | Reduced migration rates and enhanced healthcare quality | Improving working conditions, providing training opportunities | Economic instability, lack of job satisfaction, better opportunities abroad | Doctors and nurses | Address root causes of migration | Ministry of Health, Healthcare Sector | National | Ensafnews | Why are Iranian doctors looking for job opportunities abroad?(143) | 13 |
| Lack of replacement for emigrating doctors | Decreased dissatisfaction among medical professionals/ Provides clearer statistics and policies | Reduce restrictions on residency exams and licensure | Economic and social factors, political instability, lack of job opportunities | Doctors and medical workforce | Evaluate accuracy of migration statistics | Medical Council of Iran | National | Farhikhtegan | Doctors' migration: From reality to exaggera tion (144) | 14 |
| High costs of implementation, administrative challenges | Reduced migration, improved healthcare access | Financial support, policy reforms | Financial issues, inflation, lack of motivation, and job insecurity | Doctors and healthcare workers | Address doctor migration challenges | Ministry of Health, Medical Council | National | Serat News, IMNA | Why Doctors Are Migrating from Iran(145) | 15 |
| Long-term implementation required | Better regional retention of doctors | Provide equitable educational opportunities | Economic and social factors, lack of job satisfaction, better opportunities abroad | Medical students, rural areas | Address inequities in education | Education Sector, Ministry of Health | National | Abdolmajid Sheikhi (Economist) | Impact of Educational Inequality on Medical Workforce(145) | 16 |
| Requires systemic changes, long-term commitment, budgetary constraints | Reduced migration rates, improved healthcare access/ better resource allocation | Structural reforms, financial incentives, policy changes | Economic and social factors, lack of job opportunities, political instability | Healthcare professionals (Doctors, Nurses, Students) | Address economic, social, and governance issues causing migration | Ministry of Health, Medical Universities | National/International | Dr. Ali Akbar Haghdoost and Team, IRNA, WHO References | Migration of Doctors and Nurses: Causes and Implications(71) | 17 |
| High taxation, mismanagement of key specialties, and reduced mutual understanding between professional and public sectors | Increased doctor migration, declining morale, and reduced enrollment in critical specialties. | Offer financial incentives, rebuild trust between doctors and society, and promote doctors' contributions while regulating malpractice. | Economic and social factors, lack of job satisfaction, better opportunities abroad | Doctors and healthcare specialists | Reduce migration and retain specialized professionals in the healthcare sector. | Ministry of Health, Medical Academy of Iran | National | Dr. Alireza Marandi (President of the Medical Academy) | Why Are Doctors Migrating?(64) | 18 |
| Income disparity, lack of applicants in critical specialties, and diminished social respect for doctors. | Growing doctor shortage, reliance on foreign doctors, and declining healthcare quality. | Provide financial incentives, address income and workload disparities, and improve media portrayal of doctors. | Economic and social factors, political instability, lack of job opportunities | Doctors and healthcare specialists | Reduce migration and stabilize the healthcare workforce. | Ministry of Health, Medical Council | National | IMNA and Economic Daily Report | Why Did 16,000 Doctors Migrate from Iran?(65) | 19 |
| Increased workloads for remaining doctors, long training cycles for replacements, reliance on foreign staff if migration persists | Stabilized healthcare workforce, reduced reliance on foreign medical staff, and maintained quality of healthcare services | Provide financial, social, and technological support to retain doctors | Economic and social factors, lack of job satisfaction, better opportunities abroad | Healthcare professionals | Retain medical professionals and reduce migration | Ministry of Health, Medical Council | National | Dr. Hossein Kermanpour, Sina Hospital | Doctors Take Their Genes and Migrate(146) | 20 |
| High implementation costs, Short-term ineffectiveness | Potential improvement in healthcare quality if issues are resolved; retention of skilled professionals. | Discussion with government officials to address migration issues and improve conditions for medical professionals. | Economic instability, Resource constraints and limited access to equipment in some healthcare sectors, and better opportunities abroad. | Iranian Physicians | To discuss the reasons and impacts of physician migration from Iran | Medical Council of Iran | National/Interview | Mohammad Reza Zafarghandi, former head of the Medical Council of Iran | What is the reason for the increase in the immigration of Iranian doctors?(66) | 21 |
| Legal challenges regarding the constitutionality of the policy; potential exclusion of qualified students from training programs due to inability to meet collateral requirements. | Restriction on medical students' ability to continue their education if they cannot provide collateral; potential decrease in the number of trained professionals. | Implementation of two types of collateral for residency students: a notarized commitment with two non-retired employee guarantors or a property guarantee. | Economic instability, lack of job satisfaction, better opportunities abroad | Medical students, residents | Prevent physician migration | Ministry of Health, Medical Universities | National/Policy | Ministry of Health | Strange directive from the Ministry of Health to prevent the migration of doctors(67) | 22 |
| Slow implementation of solutions, cultural/legal barriers, bureaucracy, and conflicting policies leading to continued brain drain and inequality in healthcare. | Reduced negative effects of emigration, improved access to expertise, economic growth, national development. Improved collaboration with international experts, enhanced national development | Promote brain circulation through expert participation in education, research, and entrepreneurship, foster patriotism, and offer honorary roles and remote opportunities. | Economic instability, social tensions, organizational inefficiencies, and attraction of better opportunities abroad. | Experts, academics, and those interested in migration, specifically in the health field. | To discuss the migration of experts, particularly in the health sector, and explore solutions such as brain circulation. | Future Research Institute in Health, Kerman University of Medical Sciences | Report of an expert meeting | Farhangestan of medical sciences | Report of the First Expert Meeting on the Migration of Experts with a Focus on Brain Circulation (89) | 23 |
| - 10+ years needed to see significant improvement - Potential reduction in quality due to increased medical school admissions | - Reduction in rural health inequities - Enhanced physician retention - Potentially improved healthcare outcomes in underserved areas - Improved medical education policies - Potential to address long-term healthcare needs | - Increase medical education capacity (Program 7 Development Law) - Provide better facilities in remote areas - Offer financial and non-financial incentives - Improve public and workplace safety for healthcare workers - Enhance distribution of physicians across the country | - Low income - Lack of facilities in remote areas - Poor working conditions - Limited professional development opportunities | Iranian physicians and medical system | Addressing the shortage and distribution of physicians | Iranian Medical Council | Report | Leila Shoghi | Healthcare Crisis in Underprivileged Areas(48) | 24 |
| - High upfront costs - Challenges in aligning short-term and long-term strategies | - Stabilized distribution of doctors across Iran - Reduced gaps in critical specialties | - Provide equitable incentives for doctors in underserved areas - Ensure healthcare access aligns with regional population distributions - Better income and professional recognition for doctors | - Unequal distribution of doctors - High migration rates - Poor infrastructure in rural areas | Physicians across different specialties | To identify gaps in physician distribution and explore solutions for retention | Deputy Director of Technical and Supervisory Affairs of the Medical System Organization | Report | Dr. Reza Laripour, 2024 | Observational commentary and official statistics(48) | 25 |
| - Expensive reform measures - Risk of brain drain to private practice despite interventions | - Reduction in physician dropout rates from clinical fields - Improved patient outcomes in underrepresented fields | - Increase recognition of healthcare workers’ challenges - Introduce policy reforms targeting physician satisfaction - Strengthen legal protections for healthcare workers | - Rising migration numbers among specialists (e.g., anesthesiologists, pediatricians) - Internal migration to non-clinical fields | Iranian healthcare workers | To evaluate migration trends among Iranian physicians | Sharif university | Report | Sharif Migration Observatory, 2024 | Observational data from migration monitoring system(68) | 26 |
| Difficulty in securing sufficient funding, resistance to changes in economic policies | Improved retention of professors in academia | Establishing economic support committees, linking universities to industries, finding alternative funding | Low salaries, lack of economic incentives, housing issues | Professors, academic staff | Addressing economic issues to reduce professor migration | Universities, Ministry of Science | Official Statement | Hossein Simayi Sarraf/ Minister of Science's Report | Migration of 25% of Professors: Efforts to Solve Economic Issues(30) | 27 |
| Balancing increased capacity with infrastructure upgrades, funding challenges | Enhanced retention of skilled personnel/ Improved healthcare workforce distribution | Strengthening welfare systems, enhancing training facilities, increasing wages, revising policies | Poor infrastructure, inadequate welfare, low wages, limited career growth | Medical students, young physicians | Retain young medical professionals and prevent migration | Medical universities | Official Statement | Masoud Habibi/ Statement by Deputy Minister of Health | Improving Welfare for Medical Students(147) | 28 |
| Coordination among relevant organizations, administrative delays, and sustainability of solutions. High implementation costs, resistance to change, difficulty in monitoring and evaluating effectiveness. | Reduced brain drains, improved national development, and strengthened global scientific collaboration. | -Improve recruitment  -Support talented individuals  -Promote research capacity  -Expand graduate training  -Strengthen cultural values  -Elevate experts’ status  - Foster collaboration  - Enable entrepreneurship | Lack of career opportunities, inadequate recognition, insufficient facilities, and weak national connections. | Elites and Specialists | Reduce the migration of elites and specialists from the country. | Ministry of Science, Research and Technology; Ministry of Health | Policy Document | Supreme Council of Cultural Revolution | Policies and Strategies to Reduce Elite Migration(148) | 29 |
