# Supplementary material for: Determinants and policy approaches to healthcare professional retention in Iran: A mix of scoping review and qualitative evidence
Source: PLoS One. 2026 Apr 21;21(4):e0339855. doi: 10.1371/journal.pone.0339855 (PMC13099093; doi:10.1371/journal.pone.0339855)
Supplement: S3 Table — (DOCX) [file pone.0339855.s003.docx]

Table 3: Themes, Subthemes, and Supporting Evidence on Health Workforce Retention in Iran

| **Category** | \| **Main Theme** \| \| --- \|  \|  \| \| --- \|  \|  \| \| --- \| | **Subtheme** | **Emergent Themes** | **Representative Quotes (Migrated Health Workers)** | **Supporting Evidence (Health Officials)** |
| --- | --- | --- | --- | --- | --- | --- | --- | --- |
| **Reasons for Migration** | Economic Factors | Low salaries and purchasing power | - Economic instability - Lack of financial predictability | *Given the economic fluctuations and uncertainty, it was difficult to plan for a stable life, so I chose to migrate for better prospects for my family* | *“Salary scales in the health sector do not currently match those of many destination countries, making retention more difficult.”* |
|  |  | \| Economic instability and inflation \| \| --- \|  \|  \| \| --- \| | - High taxes - Rising inflation | *“Low and rather unstable wages combined with the higher inflation made me have to leave.”* | *Inflation and economic pressures have influenced the decisions of some professionals to seek work abroad* |
|  | \| Professional Dissatisfaction \| \| --- \|  \|  \| \| --- \| | Limited professional and research opportunities | - Limited funding for research - Lack of equity in promotions | *Sanctions and the reduction of the budgets of universities have limited the availability of well-equipped laboratories or the latest scientific equipment. This has left many professors concerned that they are being left behind in the global scientific revolution and that they must emigrate in order to keep up.* | *“Funding for research and education needs to expand to strengthen retention of skilled academics.”* |
|  |  | \| High workload and burnout \| \| --- \|  \|  \| \| --- \| | - Overwork and stress - Insufficient support systems | *"The patient flow was so high that it was quite challenging to maintain personal or professional interest.”* | \| *"Our hospitals and universities are short on funding for innovative projects and advanced research."* \| \| --- \|  \|  \| \| --- \| |
|  | \| Governance and Structural Barriers \| \| --- \|  \|  \| \| --- \| | \| Management and policy inefficiencies \| \| --- \|  \|  \| \| --- \| | - Limited transparency  - Need for structural reform | *"The healthcare system needs to be reformed to address the root causes of the problems."* | \| *“Governance inefficiencies may reduce confidence in institutions and affect motivation.”* \| \| --- \|  \|  \| \| --- \| |
|  |  | \| Limited merit-based recognition \| \| --- \|  \|  \| \| --- \| | - Need for fair evaluation  - Transparent promotion criteria | *“The absence of a merit award system and recognition for hard work was quite demotivating.”* | *Academic systems benefit from transparent and performance-based promotion structures* |
|  |  | \| Fragmented policy leadership \| \| --- \|  \|  \| \| --- \| | Divided responsibilities  - Coordination challenges | *“Overlapping responsibilities among organizations make the system less efficient.”* | \| *"Policymaking leadership is fragmented and creates confusion and inefficiencies in the healthcare system."* \| \| --- \|  \|  \| \| --- \| |
|  | \| Educational Challenges \| \| --- \|  \|  \| \| --- \| | \| Quota-based admissions \| \| --- \|  \|  \| \| --- \| | - Perceived inequities in admission | *The unequal admission standards are against the principle of meritocracy and demotivate hard-working students.* | \| *“Ensuring fairness in admissions can improve motivation and trust in educational institutions.”* \| \| --- \|  \|  \| \| --- \| |
|  |  | \| Centralization of resources \| \| --- \|  \|  \| \| --- \| | *- Unequal distribution of opportunities* | *"Many opportunities for research are concentrated in Tehran and thus other regions are ignored and population shift is encouraged."* | \| *Research budgets and research grants are usually targeted for certain and famous universities.* \| \| --- \|  \|  \| \| --- \| |
|  |  | \| Weak university autonomy \| \| --- \|  \|  \| \| --- \| | - Lack of academic freedom - Bureaucratic obstacles | *Inadequate autonomy reduces creativity and erodes academic democracy.* | *“Enhancing university autonomy could foster academic freedom and innovation.”* |
|  | \| Social and Psychological Drivers \| \| --- \|  \|  \| \| --- \| | \| Erosion of social trust \| \| --- \|  \|  \| \| --- \| | - Reduced cohesion  - Perception of limited opportunities | *“Many professionals feel uncertain about their future and seek stability abroad.”* | \| *"Migration has become a social norm, everyone around me is leaving, so I think it's inevitable."* \| \| --- \|  \|  \| \| --- \| |
|  |  | \| Ethical and dignity concerns \| \| --- \|  \|  \| \| --- \| | - Lack of respect for professionals | *"In a situation where dignity and civil rights are violated, people cannot help but leave their country."* | \| *"Healthcare workers are undervalued and disrespected, so they are leaving."* \| \| --- \|  \|  \| \| --- \| |
|  | \| International Pull Factors \| \| --- \|  \|  \| \| --- \| | \| Better opportunities abroad \| \| --- \|  \|  \| \| --- \| | - Job security - Respect - Superior infrastructure | *Destination countries attract migrant professors by offering social welfare and valuing science which make the conditions there attractive.* | *Healthcare systems abroad offer job security, respect, and superior infrastructure* |
| **Migration Process** | International educational and research opportunities | Academic and professional sponsorships | Fellowships - Specialized migration programs | *“I got a fellowship in the U.S. and that was the beginning of my migration.”* | *"(The US, Canada and Germany among others) recruit skilled workers and offer residency and citizenship options."* |
|  | Challenges of professional and cultural adaptation | Regulatory barriers - Language and cultural adaptation | - Professional and cultural adaptation | *“The biggest difficulty was the change in the healthcare system and the regulations of the new country.”* | *"Language barriers are a big issue initially, as well as cultural differences."* |
| **Advantages of Migration** | Improved Career Opportunities | Access to resources - Professional respect - Work-life balance | - Advanced technology - Encouraging workplace atmosphere | *“I am in Canada now and I have been able to maintain a good balance between my professional and personal life and also earn a higher income.”* | *"Healthcare systems abroad offer job security, respect, and superior infrastructure."* |
| **Challenges of Migration** | Family Separation and Cultural Adjustment | - Homesickness - Cultural and emotional barriers | - Emotional barriers - Family separation | *“For me the biggest challenge has been the long periods of time away from my family.”* | *"Living away from the culture and family is emotionally draining, even after years."* |
| **Impacts of Migration on systems** | Short-Term Impacts | Healthcare workforce shortages | - Workforce gaps - Underserved areas | *“A shortage of medical professionals results in critical gaps in the workforce, especially in depleted areas”* | *"The departure of skilled professionals has adverse effects on efficiency in key sectors."* |
|  |  | Economic disruption | - Reduced productivity - Inefficiencies | *"Deficiency of labor returns affects the economic growth and productivity negatively.”* | *"The departure of talented people affects the country's capacity to innovate and participate in international research."* |
|  | Long-Term Impacts | Decline in intellectual capital | - Loss of talent - Reduced innovation | *"The departure of cultural and academic leaders also limits Iran's role in the international intellectual activities"* | *"Participants emphasized that large-scale out-migration of skilled professionals shifts talent toward high-income countries and can worsen disparities…."* |
|  |  | Reduced cultural and academic leadership | - Fewer mentors - Fewer knowledge hubs | *“Losing experienced leaders limits local academic growth.”* | *“The migration of educators and researchers affects mentorship and training.”* |
| **Effectiveness of Current Policies** | Limited Impact of Retention Policies | Insufficient financial incentives | - Inadequate bonuses - Lack of root cause solutions | *“Government provided bonuses do not address the source of migration.”* | *"Financial incentives are not enough to keep skilled professionals."* |
|  |  | Lack of cohesive national strategy | - Fragmented policies - No unified vision | *“Retention or engagement strategies are not cohesive, with no clear strategy to keep or attract talent.”* | *"There is no cohesive national strategy which is a result of poor retention efforts."* |
|  | Restrictive Measures Backfiring | Penalties for emigration create resentment | - Mandatory service obligations - Increased dissatisfaction | *“Compulsory service terms and penalties adverse to skilled professionals”* | *"Restrictive measures for example, penalties for emigration only serve to exacerbate dissatisfaction and promote the exit of professionals."* |
| **Solutions to Reduce Migration** | Financial Incentives | -Competitive salaries - Funding for research - Tax incentives | - Improved salaries - Housing support | *“As for the government, had it provided more financial support to research, I might have stayed.* | *"Paying salaries of a minimum of $1,500 per month as in Turkey may help in retaining talent."* |
|  | Organizational Improvements | Organizational reforms - Improved working conditions | - Transparency - Career development plans | *It is important that university positions are provided to qualified persons. This will also help to restore the accuracy of the universities and to prevent the migration of elites.* | *The university should not be compared to a military barracks. In any case, professors should be allowed to constructively criticize policies and management and offer suggestions for improvement. Scientific criteria should be used for recruiting professors instead of ideological criteria. This will not only enhance the quality of education but also decrease the reason to emigrate.* |
|  | Systemic Reforms | Institutional improvement | - Policy stability  - Governance efficiency | *"The healthcare system needs fundamental changes to address the source of the problems."* | *"Power in higher education is shared among different actors, which results in ineffectiveness and misunderstandings."* |
|  | Recognition and Support | Appreciation of expertise | Acknowledgment of achievements - Tangible rewards | *“The recognition of our achievements would make us want to stay and contribute to the community.”* | *"Healthcare workers have to be appreciated and respected in their position."* |
|  | Administrative Simplification | Bureaucracy reduction | - Streamlined procedures | *“Excessive paperwork slowed progress.”* | *“Simplifying administrative systems supports efficiency.”* |
| **Reverse Migration Solutions** | Incentives for Returning Professionals | - Financial and professional incentives - Research autonomy | - Competitive pay  - Advanced facilities | *“Providing adequate facilities and salaries would attract many professionals back.”* | *“A stable economic future will make the elites to consider returning.”* |
|  | Academic and Professional Reintegration | - Reintegration challenges - Credential recognition | - Clear pathways for reintegration - Recognition of international qualifications | *"It would be easier to come back if there were well-defined paths to re-establish themselves in academia or healthcare."* | *"There is a need for programs that recognize the global experience."* |
|  | Collaboration Opportunities | - International joint projects - Academic partnerships | - Joint research projects - Exchange programs | *An inverse migration can be encouraged by joint research projects between Iran and international universities.* | *"Creating a Diaspora Engagement Office can help to foster partnership and business alliances with expatriates."* |
|  | Improved Research Facilities | - Lack of modern research tools - Inadequate infrastructure | -Advanced laboratories - Modern medical technologies | *"Having access to the latest research equipment in Iran would make me consider returning."* | *If we want to prevent the migration of elites, we have to work collectively across media, lifestyle, economic, and resource management.* |
|  | Opportunities for Innovation | -Lack of innovation culture - Limited freedom in research | - Academic freedom - Innovation platforms | *I would be happy to lead a project if a platform is provided to me to execute innovative ideas* | *"Let expatriates participate in international conferences and research to increase the link with Iran."* |
