# Supplementary material for: Determinants and policy approaches to healthcare professional retention in Iran: A mix of scoping review and qualitative evidence
Source: PLoS One. 2026 Apr 21;21(4):e0339855. doi: 10.1371/journal.pone.0339855 (PMC13099093; doi:10.1371/journal.pone.0339855)
Supplement: S4 Table — (DOCX) [file pone.0339855.s004.docx]

Table 4: Annual Publication Counts on the Retention of Iranian Health Personnel

| **Year** | \| **Number of Articles** \| \| --- \|  \|  \| \| --- \| | \| **References** \| \| --- \|  \|  \| \| --- \| |
| --- | --- | --- | --- | --- | --- | --- |
| 2005 | 1 | (124) |
| 2010 | 1 | (95) |
| 2013 | 1 | (94) |
| 2014 | 1 | (72) |
| 2016 | 1 | (19) |
| 2018 | 6 | [9], [24], (26) [47], [67], [81] |
| 2019 | 2 | (86)(73) |
| 2020 | 2 | (55)(82) |
| 2021 | 3 | (25) (28)(132) |
| 2022 | 6 | (18) (29)(34)(87)(83)(137) |
| 2023 | 11 | (16,24,46,47,69,76,81,93,119,123), (139) |
| 2024 | 6 | (31)(74)(138)(75)(77)(60) |
| 2025 | 2 | (36)(38) |
