# Supplementary material for: Determinants and policy approaches to healthcare professional retention in Iran: A mix of scoping review and qualitative evidence
Source: PLoS One. 2026 Apr 21;21(4):e0339855. doi: 10.1371/journal.pone.0339855 (PMC13099093; doi:10.1371/journal.pone.0339855)
Supplement: S5 Table — (DOCX) [file pone.0339855.s005.docx]

Table 5: Frequency of Retention Factors Reported in the Articles

| Factor | Number of Articles | References |
| --- | --- | --- |
| Economic | 23 | (2), (3), (94), (17), (25), (9) (26), (29), (30), (31), (33), (86), (73), [10], (46), (87), (83), (74), (81), (95), (84) (36)(38) |
| Political | 11 | (3), (17), (16) , (46) (83) (74) (123), (95), (84), (36)(38) |
| Organizational/Administrative | 16 | [1], [9], [14], [16], [18], (26) [28], [30], [50], [69], [80], [81], [83], [84], [91], [150] |
| Social/Cultural | 14 | \|  \| \| --- \|  \| [1], [2], [16], [21], (28), [30], [50], [78], [80], [81], [91], [92], (36)(38) \|  \| \| --- \| --- \| |
| Educational | 7 | (8,17,32–34,55,84) |
| Structural | 3 | (3), (12)(46) |
| Occupational | 2 | (3), (46) |
| Psychological | 3 | [1], [10], [13] |
