# Supplementary material for: Determinants and policy approaches to healthcare professional retention in Iran: A mix of scoping review and qualitative evidence
Source: PLoS One. 2026 Apr 21;21(4):e0339855. doi: 10.1371/journal.pone.0339855 (PMC13099093; doi:10.1371/journal.pone.0339855)
Supplement: S6 Table — (DOCX) [file pone.0339855.s006.docx]

Table 6: Comparative Evaluation of Health Workforce Retention Policies in Iran Across Four Key Dimensions: Effectiveness, Coverage, Cost-efficiency, and Sustainability.

| Policy/Program | Effectiveness | Coverage | Cost-efficiency | Sustainability |
| --- | --- | --- | --- | --- |
| Research Development Programs | Moderate: Helped retain a few researchers | Low (7% of eligible) | Low (high cost per capita) | Weak (year-to-year budget fluctuations) |
| Physician-Researcher Initiative | Low-Moderate: conceptually appealing | Very Low (early-stage and focused on specific institutions) | Low (requires structured funding and institutional buy-in.) | Weak (pilot-dependent) |
| Financial Retention Packages (Hardship Allowances) | Mixed: improved short-term retention but sensitive to inflation and currency fluctuations. | Medium (implemented across multiple sectors.) | Low (effectiveness depends on timely adjustment to living costs.) | Low (not inflation-adjusted) |
| Geographic Compulsory Service | Ineffective: high resistance, low motivation | High (nationwide implementation across all provinces.) | Cost-unclear (potentially efficient due to existing infrastructure.) | Very Weak (pushes long-term migration) |
| Diaspora Engagement (Short-term fellowships) | Promising potential, minimal implementation | Very Low | High potential return on investment through capacity building. | Moderate (requires coordination) |
| Equity in HR Distribution & Organizational Justice | Minimal: partial efforts, limited local adaptation | Medium (adopted in select hospitals and universities.) | Medium (administrative resources required but yields intangible benefits in morale and commitment.) | Weak (long-term success relies on embedding fairness principles into HR governance.) |
| Health Tourism Initiative | Moderate-High: Potential to generate revenue for system modernization and create high-skill jobs, indirectly boosting retention | Low-Moderate: Currently concentrated in major private centers in Tehran and a few other cities | High: Generates direct foreign currency revenue; initial infrastructure investment can be offset by returns. | High: Market-driven and aligns with national economic diversification goals; can create a self-sustaining cycle of investment in healthcare. |
