# Supplementary material for: Determinants and policy approaches to healthcare professional retention in Iran: A mix of scoping review and qualitative evidence
Source: PLoS One. 2026 Apr 21;21(4):e0339855. doi: 10.1371/journal.pone.0339855 (PMC13099093; doi:10.1371/journal.pone.0339855)
Supplement: S7 Table — (DOCX) [file pone.0339855.s007.docx]

Table 7: Assessment of Retention and Reverse Migration Strategies in Iran Based on Feasibility, Effectiveness, and Sustainability

| Strategy Category | Feasibility | \| **Effectiveness** \| \| --- \| | Sustainability | Explanation |
| --- | --- | --- | --- | --- | --- |
| 1. Economic & Professional Incentives | Medium | High | Medium-Low | Requires stable funding; directly addresses top migration drivers; vulnerable to inflation. |
| 2. Governance Reforms | Low-Medium | High | High (if structural) | Politically sensitive; strong impact on fairness; sustainable if institutionalized. |
| 3. Diaspora Engagement | Medium-High | Medium | High | Technically feasible; evidence from other countries; low-cost and long-term benefits. |
| 4. Social & Cultural Strategies | High | Medium | High | Easily implementable; boosts morale; sustainable through media and education. |
| 5. International Collaboration | Low-Medium | High (if viable) | Medium | Dependent on diplomacy and sanctions; expands opportunities and networks. |
| 6. Educational Reform | Medium | High | High | Needs policy alignment; matches global trends; long-term structural change. |
| 7. Technological Innovation | Medium | Medium-High | High | Moderate investment needed; enhances service delivery; scalable nationwide. |
| 8. Quality of Life Improvements | Medium-Low | High | Medium | Cost-intensive; targets non-professional push factors; dependent on urban planning. |
| 9. Decentralized Strategies | High | High | High | addresses context-specific challenges |
| 10. Participation in Decision-Making | High | High | High | directly addresses feelings of disempowerment |
| 11. Circular Migration Models | High | High | High | reduces the psychological and logistical barriers of permanent return |
| 12. Professional Community Building | High | High | High | fostering a sense of belonging and shared mission |
