# Supplementary material for: Determinants and policy approaches to healthcare professional retention in Iran: A mix of scoping review and qualitative evidence
Source: PLoS One. 2026 Apr 21;21(4):e0339855. doi: 10.1371/journal.pone.0339855 (PMC13099093; doi:10.1371/journal.pone.0339855)
Supplement: S1 Appendix — (DOCX) [file pone.0339855.s008.docx]

**Appendix A – Semi-Structured Interview Guide**

**Section 1: Introduction and Consent**

- Can you briefly introduce yourself (profession, years of experience, current country of residence)?
- Have you read and agreed to the informed consent form?

**Section 2: Migration Motivation (Push and Pull Factors)**

**Main Questions:**

1. What were your main reasons for considering migration (or What are the main reasons for elite migration)?
2. What factors influenced your final decision to migrate?

**Section 3: Experiences Abroad (for emigrated professionals only)**

**Main Questions:**

3. How do you compare your current working conditions with those in Iran?

4. What challenges did you face when adapting to the new system?

**Probe Examples:**

- Workload, pay, workplace culture?
- Integration, recognition of credentials, language barriers?

**Section 4: Systemic Consequences and Reflections**

**Main Questions:**

5. In your opinion, what are the impacts of health workforce migration on Iran’s healthcare system?

6. How do you evaluate current Iranian policies addressing migration?

**Section 5: Future Outlook and Policy Suggestions**

**Main Questions:** 7. What measures or policies do you think would help retain healthcare professionals in Iran?

8. Would you consider returning to Iran? Under what conditions?

**Section 6: Closing**

1. Is there anything else you would like to share about your experience or perspectives on health worker migration?
